# Immunotherapy-resistant acute lymphoblastic leukemia cells exhibit reduced CD19 and CD22 expression and BTK pathway dependency

**DOI:** 10.1172/JCI175199

**Published:** 2024-02-20

**Authors:** Sarah Aminov, Orsi Giricz, David T. Melnekoff, R. Alejandro Sica, Veronika Polishchuk, Cristian Papazoglu, Bonnie Yates, Hao-Wei Wang, Srabani Sahu, Yanhua Wang, Shanisha Gordon-Mitchell, Violetta V. Leshchenko, Carolina Schinke, Kith Pradhan, Srinivas Aluri, Moah Sohn, Stefan K. Barta, Beamon Agarwal, Mendel Goldfinger, Ioannis Mantzaris, Aditi Shastri, William Matsui, Ulrich Steidl, Joshua D. Brody, Nirali N. Shah, Samir Parekh, Amit Verma

**Affiliations:** 1 Department of Oncology, Blood Cancer Institute, Montefiore Einstein Comprehensive Cancer Center, Bronx, New York, USA.; 2 Hematology and Medical Oncology, Icahn School of Medicine at Mount Sinai, New York, New York, USA.; 3 Pediatric Oncology Branch, Center for Cancer Research and; 4 Laboratory of Pathology, Center for Cancer Research, National Cancer Institute, Bethesda, Maryland, USA.; 5 Department of Pathology, Albert Einstein College of Medicine, Bronx, New York, USA.; 6 Department of Medicine, Division of Hematology/Oncology, Hospital of University of Pennsylvania, Philadelphia, Pennsylvania, USA.; 7 GenomeRxUS LLC, Philadelphia, Pennsylvania, USA.; 8 Department of Oncology, Dell Medical School, University of Texas at Austin, Austin, Texas, USA.

**Keywords:** Oncology, Cancer

## Abstract

While therapies targeting CD19 by antibodies, chimeric antigen receptor T cells (CAR-T), and T cell engagers have improved the response rates in B cell malignancies, the emergence of resistant cell populations with low CD19 expression can lead to relapsed disease. We developed an in vitro model of adaptive resistance facilitated by chronic exposure of leukemia cells to a CD19 immunotoxin. Single-cell RNA-Seq (scRNA-Seq) showed an increase in transcriptionally distinct CD19^lo^ populations among resistant cells. Mass cytometry demonstrated that CD22 was also decreased in these CD19^lo^-resistant cells. An assay for transposase-accessible chromatin with sequencing (ATAC-Seq) showed decreased chromatin accessibility at promoters of both CD19 and CD22 in the resistant cell populations. Combined loss of both CD19 and CD22 antigens was validated in samples from pediatric and young adult patients with B cell acute lymphoblastic leukemia (B-ALL) that relapsed after CD19 CAR-T–targeted therapy. Functionally, resistant cells were characterized by slower growth and lower basal levels of MEK activation. CD19^lo^ resistant cells exhibited preserved B cell receptor signaling and were more sensitive to both Bruton’s tyrosine kinase (BTK) and MEK inhibition. These data demonstrate that resistance to CD19 immunotherapies can result in decreased expression of both CD19 and CD22 and can result in dependency on BTK pathways.

## Abstract 2

Acute lymphoblastic leukemia cells resistant to CD19 immunotherapies exhibit combined loss of CD19 and CD22 and an enhanced sensitivity to BTK and MEK inhibition.

## Graphical abstract

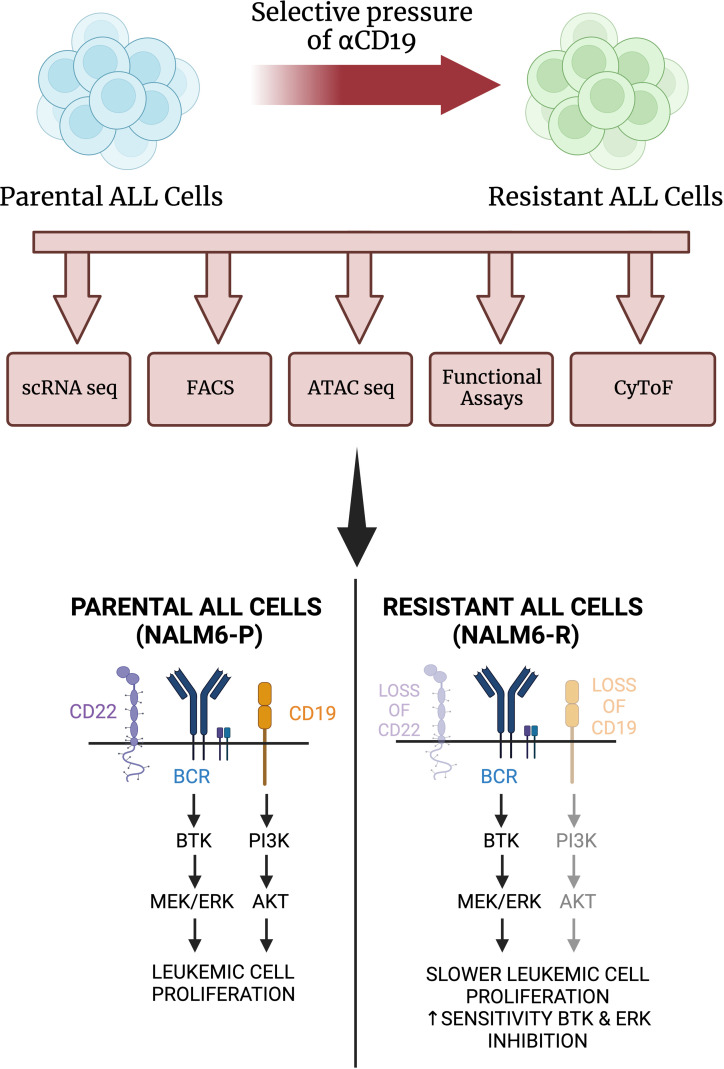

## Introduction

Although much progress has been made in the treatment of B cell acute lymphoblastic leukemia (B-ALL), relapsed and refractory disease is an ongoing problem. The development of CD19 chimeric antigen receptor T cell (CAR-T) therapy and other CD19 immunotherapies has dramatically improved the response rates in patients with B-ALL (1). Unfortunately, the emergence of resistant cell populations with low expression of CD19 account for approximately 30%–40% of patients with relapsed ALL after CAR-T treatment (1–3). Loss of surface CD19 on malignant cells leads to reduced efficacy of CD19-targeted CAR-Ts as well as of bispecific antibodies (4, 5). Understanding how resistance occurs as well as the cellular signaling that takes place in the resistant cell populations are critical for targeting relapsed disease after CAR-T treatment.

A potential strategy to treat patients with CD19-resistant B-ALL is to evaluate therapeutics targeting the CD22 B cell antigen (6, 7). Whether these 2 surface receptors are coregulated in malignant cells has not yet been fully elucidated. Another potential therapeutic target in B cell malignancies is the B cell receptor (BCR) pathway. Bruton’s tyrosine kinase (BTK) is associated with the BCR, and BTK inhibitors have been approved for use in chronic lymphocytic leukemia (CLL) and mantle cell lymphomas but have not been rigorously evaluated in resistant B-ALL (8). While CD19 and BCR independently regulate proliferative signaling pathways, downregulation of each one has been shown to upregulate or maintain expression of the other (9, 10). We developed an in vitro model of adaptive resistance to CD19 immunotherapies and then used it to determine whether BTK signaling would be preserved in CD19-resistant B-ALL cells. We show that inhibiting this pathway could be an avenue for CD19-targeted, immunotherapy-resistant cell populations.

## Results

### Long-term exposure to CD19 immunotoxin leads to development of resistant B-ALL cell lines.

**Figure 1:**
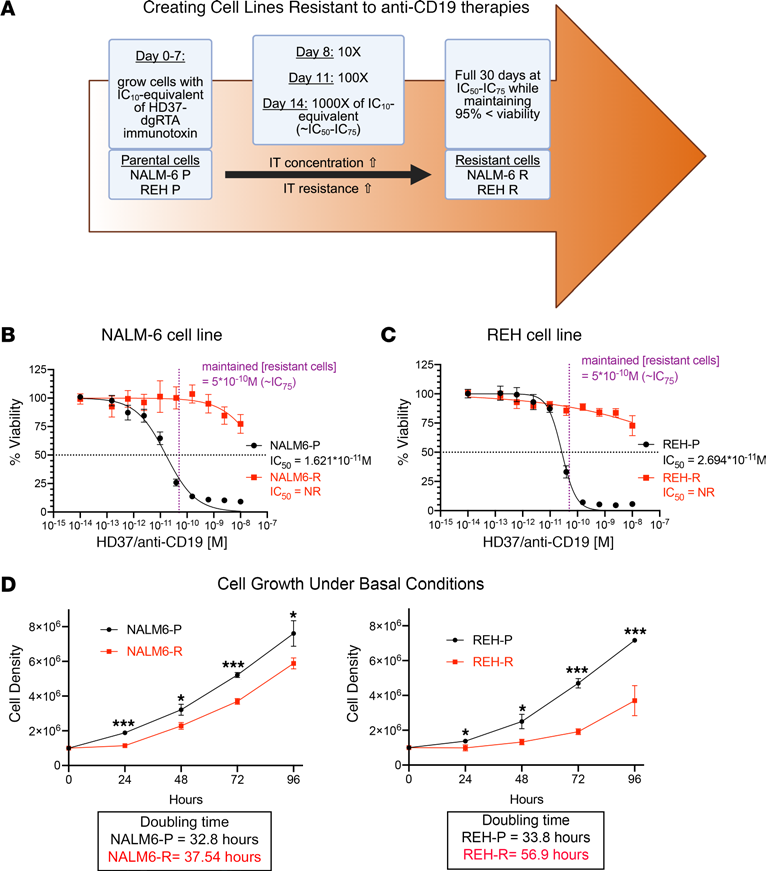
Long-term exposure to CD19 immunotoxin leads to resistant ALL cell lines. (**A**) Schema for the generation of ALL cells resistant to CD19 immunotoxin (IT) (HD37 antibody clone conjugated to the recombinant ricin B chain dgRTA), created with BioRender.com. (**B** and **C**) NALM6 and REH cell lines that were made resistant had a significantly higher IC_50_ against CD19 immunotoxin (*n* = 4). (**D**) Resistant cell lines had a slower growth rate (*n* = 3). *P < 0.05 and ***P < 0.001, by 2-tailed Student’s *t* test for analysis at each time point.

To generate B-ALL cell lines resistant to anti-CD19–based immunotherapy, parental cells of the NALM6 and REH cell lines were grown in the presence of HD37-dgRTA immunotoxin (11) at increasing concentrations over 30 days (Figure 1A). The immunotoxin is an anti–CD19 HD37 antibody clone that is conjugated to recombinant ricin B chain dgRTA (11). Once the resistant cells were generated, they were compared with the parental cells of their respective cell line for sensitivity against the CD19 immunotoxin (Figure 1, B and C). The resistant NALM6 and REH cell lines had a substantially increased IC_50_ when treated with the CD19 immunotoxin compared with the parental cells (Figure 1, B and C). Cell growth was also observed under basal conditions, in which resistant cells had a slower growth rate than did parental cells (Figure 1D).

### B-ALL cells resistant to CD19 immunotherapy have reduced CD19 expression and a distinct transcriptomic profile.

**Figure 2:**
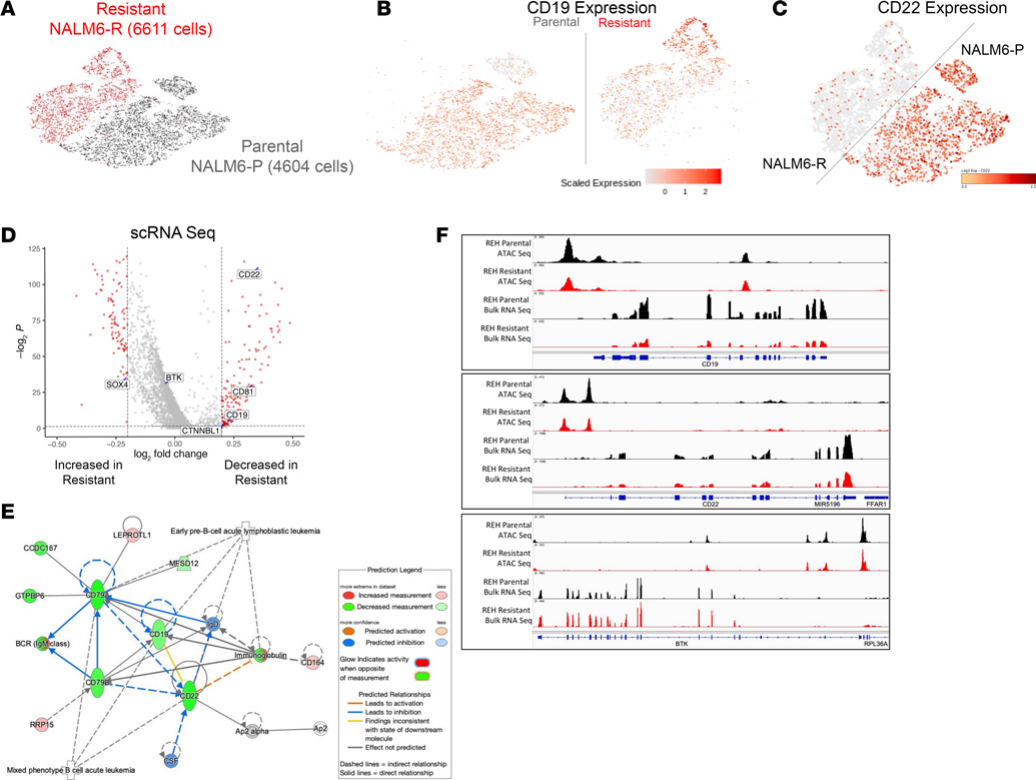
ALL cells resistant to CD19 immunotherapy have reduced CD19 and CD22 expression and a distinct transcriptomic profile. (**A**) scRNA-Seq shows that parental and resistant lines were transcriptionally distinct. (**B** and **C**) Resistant cells had an expanded population with decreased CD19 and CD22 expression. (**D**) Genes that were differentially expressed in scRNA-Seq between resistant and parental NALM6 cells. (**E**) Ingenuity Pathway Analysis for N6 scRNA-Seq. (**F**) ATAC-Seq and bulk RNA-Seq on REH parental and resistant cells showing a decrease in chromatin accessibility and expression of CD19 and CD22 and maintenance of accessibility and expression in BTK cells.

scRNA-Seq was performed to compare expression differences between NALM6-resistant and parental cell lines (Figure 2A). We analyzed a total of 6,611 NALM6 parental (NALM-P) and 4,606 NALM6-resistant (NALM-R) cells and found that the resistant and parental cells formed distinct clusters that could be further separated into CD19^hi^ and CD19^lo^ populations on the basis of transcriptomic profiles (Figure 2B). We observed that parental cells had a relatively smaller population of CD19^lo^-expressing cells, which were expanded among the resistant cells. Interestingly, NALM6-R cell types also showed decreased CD22 expression when compared with parental cells (Figure 2C). To characterize global gene expression differences in resistant NALM6 cells, we analyzed differentially expressed genes in dominant populations of parental and resistant cells (Figure 2D). Notably, we found that the expression of CD19 and CD22 was markedly decreased in the resistant cell population. We also examined genes regulating CD19 and observed an increase in the expression of *SOX4* as well as a decrease in the expression of *CTNNBL1* and *CD81*, which are CD19 activators in the resistant cells (12). When analyzing the pathways in resistant cells, we found that the BCR network was predicted to be significantly affected by transcriptomic changes (Figure 2E). Importantly, BTK expression was maintained in resistant CD19^lo^ cells. Overall, gene expression changes considerably affected T and B lymphatic pathways as well as hematological cancer networks (Supplemental Figure 2 and Supplemental Table 2; supplemental material available online with this article; https://doi.org/10.1172/JCI175199DS1).

Finally, chromatin accessibility and a correlation with gene expression were determined in REH parental and resistant cells using ATAC-Seq and bulk RNA-Seq. We observed that chromatin accessibility at both CD19 and CD22 promoter regions was decreased in resistant cells and correlated with reduced gene expression (Figure 2F). Chromatin accessibility and expression for BTK were maintained in the REH resistant cells (Figure 2F). When overall chromatin accessibility changes were analyzed via Ingenuity Pathway Analysis, we noted suggestive changes in gene networks related to cancer (Supplemental Table 3).

### Expression of both CD19 and CD22 proteins is decreased in resistant cell lines as well as in patients with clinical relapses after CD19-targeted therapy.

**Figure 3:**
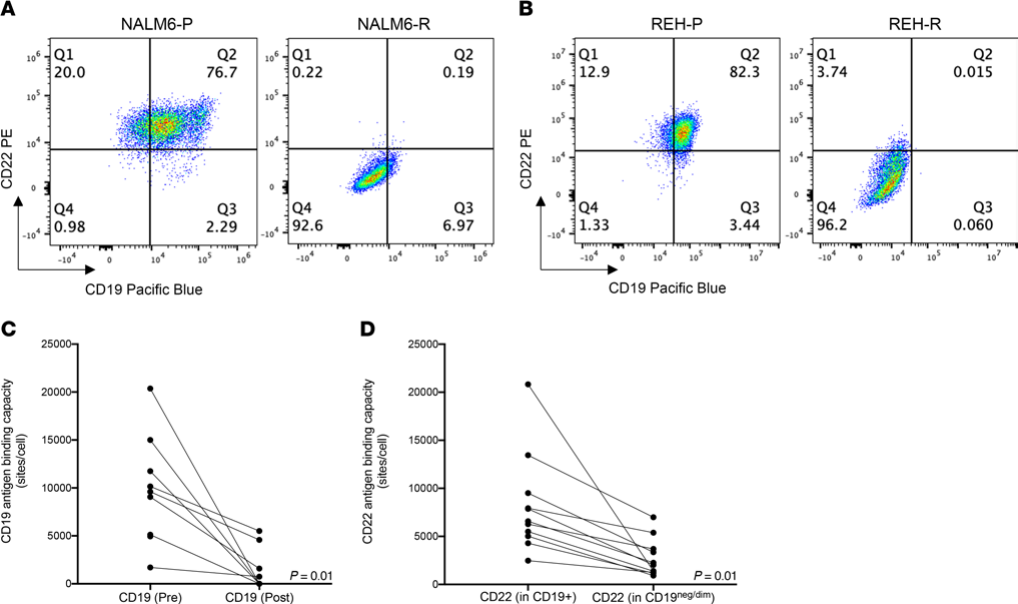
Loss of CD19 expression in CD19 CAR-T–treated pediatric patients with ALL corresponds to a loss of CD22 expression. (**A** and **B**) Reduced CD19 and CD22 expression on resistant cells seen by FACS analysis. Gating was based on the isotype control. (**C** and **D**) CD19 and CD22 antigen expression was observed in a cohort of cells from pediatric patients with ALL (*n* = 11) before treatment and after relapse following CD19 CAR-T therapy. A significant reduction of CD22 in CD19^lo^ patients after relapse was observed. The Wilcoxon signed-rank test was used for statistical analysis.

To determine CD19 and CD22 protein expression, we performed FACS analysis of both NALM6 and REH cells (Figure 3, A and B). We found that both NALM6 and REH parental cells expressed high levels of CD19 and CD22 but that these levels were decreased in the resistant cells. After observing decreased expression of both CD19 and CD22 in our model of adaptive resistance, we wanted to examine their expression in pediatric patients with ALL who had received one of several CD19-targeted therapies and had relapsed with CD19^–/dim^ disease, and who were then screened for enrollment in or received treatment in a NCI clinical trial. We examined the status of both CD19 and CD22 in a cohort of 11 patients who experienced relapse after CD19-targeted therapy. Nine of these patients received CD19-targeted CAR-T cells (2 received these cells in the context of a CD19/CD22 combinatorial CAR-T cell construct [ref. 13; ClinicalTrials.gov identifiers: NCT01593696 and NCT03448393], and 2 received blinatumomab). Six of these patients have been partially described previously (6). In this cohort, we observed that, in addition to loss/diminution of CD19 expression (Figure 3C), downregulation of CD22 expression occurred in all patients upon relapse or nonresponsiveness to therapy (Figure 3D).

### BTK expression is preserved in resistant CD19^lo^ cells.

**Figure 4:**
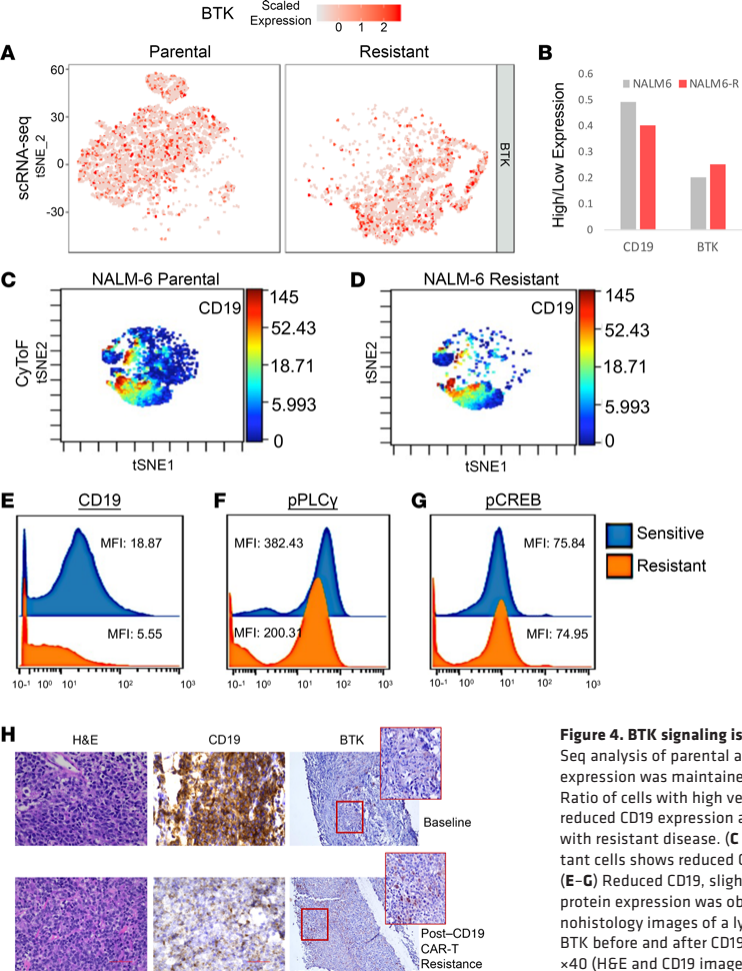
BTK signaling is active in CD19^lo^ resistant cells. (**A**) scRNA-Seq analysis of parental and resistant NALM6 cells shows that BTK expression was maintained in patients with resistant disease. (**B**) Ratio of cells with high versus low CD19 or BTK expression shows reduced CD19 expression and unaltered BTK expression in patients with resistant disease. (**C** and **D**) CyTOF analysis of parental and resistant cells shows reduced CD19 protein expression in resistant cells. (**E**–**G**) Reduced CD19, slightly reduced p-PLCγ, and preserved p-CREB protein expression was observed in resistant NALM6 cells. (**H**) Immunohistology images of a lymphoma patient’s cells stained for CD19 or BTK before and after CD19 CAR-T treatment. Original magnification, ×40 (H&E and CD19 images) and ×20 and ×40 (BTK images).

When comparing single-cell expression profiles of NALM6-P and NALM6-R cells, we found that BTK expression was not downregulated in resistant cells (Figure 4, A and B). To validate these trends with proteomics, we performed mass cytometry (cytometry by time-of-flight CyTOF]) to determine the levels of CD19 and other B cell signaling proteins in NALM6-P and NALM6-R cells (Figure 4, C and D). Consistent with the earlier transcriptional data, CD19 protein levels were reduced in resistant cells. BCR-associated phosphorylated PLCγ (p-PLCγ) and p-CREB expression was also examined and demonstrated only a slight decrease in p-PLCγ expression in resistant cells, whereas p-CREB expression was maintained (Figure 4, E–G). Finally, we obtained pre- and post-treatment samples from a patient with B cell lymphoma who was treated with CD19 CAR-T and relapsed after an initial response. Comparative immunohistochemical examination of B cell lymphoma samples at baseline and at relapse revealed a decrease in CD19 staining intensity in resistant cells with an increase in BTK intensity (Figure 4H). These data, taken together, suggest that, while CD19 was decreased in resistant cases, BCR-associated BTK expression was preserved.

### BCR dependency in anti-CD1–resistant B-ALL cells.

**Figure 5:**
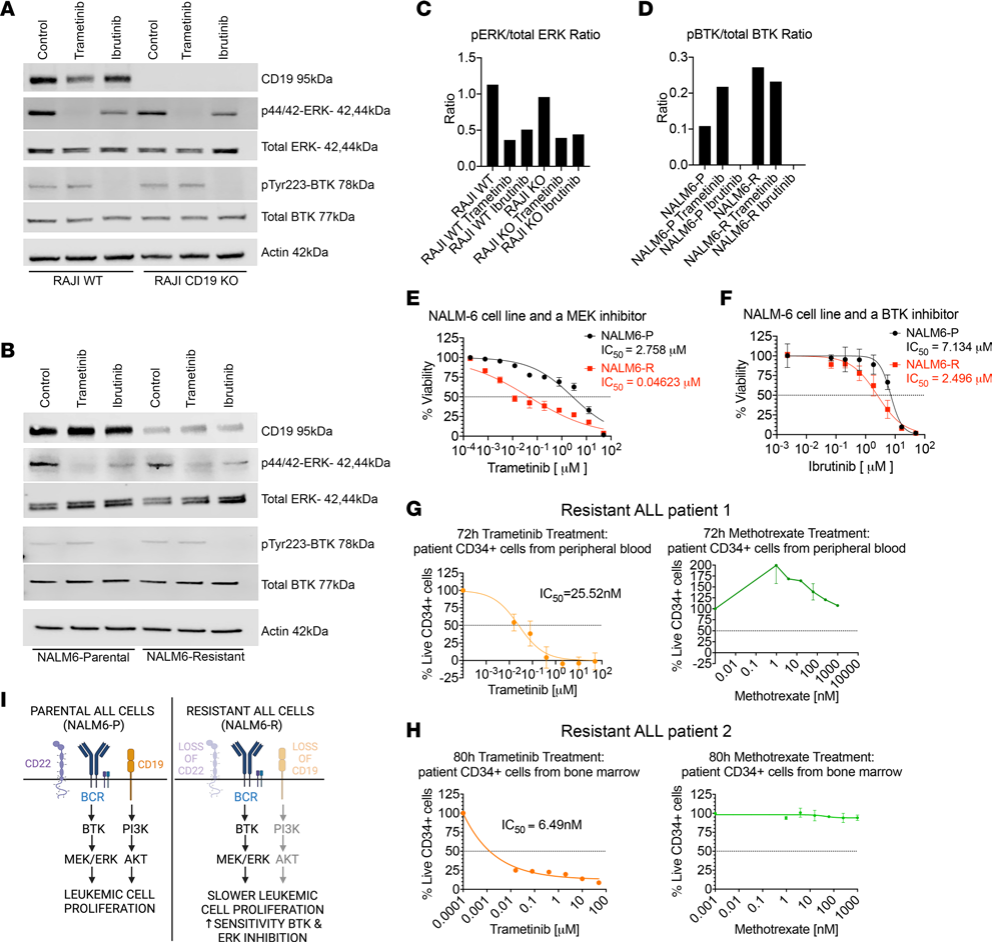
Cells resistant to CD19 immunotherapy exhibit dependency on BCR and MEK signaling. (**A** and **B**) Immunoblotting shows that ibrutinib could inhibit p-BTK in resistant cells. Resistant cells had preserved ERK activation. (**C**) p-ERK/total ERK ratio in Raji cells shows that p-ERK was maintained in Raji CD19-KO cells. (**D**) p-BTK/total BTK ratio in NALM6 cells shows that p-BTK was maintained in NALM6-R cells and that ibrutinib effectively inhibited phosphorylation. (**E** and **F**) NALM6-R cells were more sensitive to MEK and BTK inhibition (*n* = 4). (**G**) A patient with ALL resistant to CD19 immunotoxin (Resistant ALL patient 1) was sensitive to the MEK inhibitor but resistant to methotrexate (*n* = 3). (**H**) A patient with ALL resistant to blinatumomab (Resistant ALL patient 2) was sensitive to the MEK inhibitor but resistant to methotrexate (*n* = 3). (**I**) Proposed model showing that resistant ALL cells lose CD19 and CD22 expression and maintain BTK expression, leading to slower leukemic proliferation and increased sensitivity to BTK and ERK inhibition. The model was created with BioRender.com.

Since CD19 and BCR/BTK signaling are both proliferative pathways for B cell malignancies, we next sought to determine whether BCR signaling plays a functional role in CD19-resistant B-ALL. Malignant B cells (Raji) with and without CRISPR-aided CD19 KO were used, and loss of CD19 was confirmed by immunoblotting (Figure 5A). Cells with CD19 loss displayed activated/phosphorylated ERK MAPK, although the level of activation was reduced compared with WT CD19 cells (Figure 5C). Raji CD19–KO cells also showed loss of both CD19 and CD22 cell-surface expression when compared with WT cells (Supplemental Figure 1). The BTK inhibitor ibrutinib led to a decrease in activated/phosphorylated ERK in both parental and resistant cells, demonstrating that a functioning BTK was upstream of proliferative MAPK signaling. A MEK inhibitor (trametinib) was used a positive control. Immunoblots with NALM6-P and NALM6-R cells were similar to the results seen in the Raji-KO cells and demonstrated reduced CD19 and a functional BTK in resistant cells (Figure 5, B and D). Notably, NALM6-R cells had an increased p-BTK/total BTK ratio when compared with parental cells (Figure 5D). Next, NALM6 cells were treated with MEK and BTK inhibitors, and IC_50_ values were compared between parental and resistant cells (Figure 5, E and F). We observed that the resistant cells were more sensitive to each inhibitor than were the parental cells. The same trend was observed in REH parental and resistant cells as well as Raji WT and CD19-KO cells treated with the same MEK and BTK inhibitors (Supplemental Figure 3). We also observed that combination treatment with the immunotoxin and BTK inhibitor in NALM6-P cells led to additive effects on reduced cell viability (Supplemental Figure 4).

To confirm that the MEK pathway is a downstream survival pathway in CD19-resistant cells from patients, we evaluated cells obtained from patients with treatment-resistant B-ALL. One patient was resistant to the HD37-dgRTA CD19 immunotoxin (ClinicalTrials.gov identifier: NCT00450944) (Figure 5G), and the other was resistant to blinatumomab (Figure 5H), a BiTE antibody that targets CD19-expressing cells with cytotoxic T cells (14). The resistant cells were treated with the MEK inhibitor trametinib and methotrexate; both cells were insensitive to methotrexate, while retaining sensitivity to MEK inhibition.

On the basis of our results, we propose that both CD19 and the BCR work to promote leukemic cell proliferation via MEK activation in parental cells. When CD19 expression is lost, the BCR works alone to sustain proliferation. This results in slower leukemic cell proliferation and increased relative sensitivity to BTK and ERK inhibition (Figure 5I).

## Discussion

Resistance to CD19-targeted therapies is emerging as a major clinical challenge in patients with B-ALL. A sizable number of relapses occur upon downregulation of the CD19 antigen, thus reducing the efficacy of CD19-targeting CAR-T therapies, immunotoxins, and bispecific antibodies. Consideration of other avenues of treatment is necessary for these patients: specifically, targeting growth pathways that are functionally active in CD19-resistant cell populations. To explore such pathways, we developed an in vitro model of adaptive resistance based on chronic exposure to a CD19 immunotoxin. We observed that even in B-ALL cell lines, there existed a small pool of CD19^lo^ cells that expanded in resistant cells and proliferated only when CD19 was targeted. Additionally, these cells had reduced expression of previously defined CD19 activator genes (12), including *CD81* and *CTNNBL1*, and elevated expression of known inhibitors of CD19, including *SOX4*. Furthermore, chromatin accessibility at the CD19 promoter was reduced upon resistance, pointing to transcriptional downregulation of CD19 expression that caused adaptive resistance to a CD19-targeting immunotoxin.

We observed that BTK expression was maintained in resistant cells and was functionally relevant in activating downstream proliferative MEK pathways. CD19 and BCR have parallel pathways that promote cell proliferation, however, these 2 receptors have been previously shown to affect each other’s signaling pathways (15). Specifically in mantle cell lymphoma cells, the ROR1-CD19 complex was shown to effectively replace BCR/BTK signaling and promote cell proliferation (9). Alternatively, BCR signaling was shown to be enhanced in CD19-deficient primary B cells (10). The relationship between CD19 and the BCR has not yet been established in CD19-resistant B-ALL. Here, we demonstrate that BCR signaling remained intact in CD19^lo^ cell populations and that exploiting that pathway through BTK or MEK inhibition effectively inhibited the growth of CD19-resistant cells.

Interestingly, our data showed that CD22 expression decreased along with CD19 expression in resistant cells. Both CD19 and CD22 are B cell–associated antigens that emerge during pre–B cell development. It is possible that selective pressure of the CD19 immunotoxin leads to adaptive emergence of a developmentally earlier stage of cells that lack both antigens. In fact, patients who have been shown to lose CD19 during resistance have also been shown to be positive for stem/progenitor CD34 and CD123 markers, suggesting regression to a developmentally earlier stem-like state (16). Strikingly, this reduction of CD22 in addition to CD19 was observed in a set of samples from pediatric patients who were resistant to CD19-targeted immunotherapies. While some cell subsets may have preservation of CD22 expression (17), we believe our data along with others’ reports on patient cohorts in which downregulation of CD22 (6, 18) with emergence of CD19^lo^/CD19^–^ populations was seen, are of translational importance. Collectively, although CD22 immunotherapies as well as bispecific CAR-T therapies targeting both CD19 and CD22 have been used in an attempt to prevent or treat resistant patient populations (19), the concurrent downregulation of CD22 may make targeting of CD22 challenging in these cases.

Taken together, we have developed an in vitro model of resistance that can be used to determine transcriptional and signaling alterations that occur during this CD19 resistance process. Our data suggest that inhibition of the BCR and MEK pathways in patients with CD19-resistant B-ALL could be a therapeutically advantageous avenue for treatment and support further testing in clinical trials.

## Methods

### Sex as a biological variable.

Both male and female patients were included in the study, however, sex was not considered as a biological variable.

### Cell lines and human samples.

NALM6 and REH parental cell lines were purchased from the American Type Culture Collection (ATCC) and cultured according to the manufacturer’s instructions in RPMI 1640 media supplemented with 10% FBS and 1% penicillin/streptomycin. WT Raji and CD19-KO Raji (CRISPR/Cas9) cell lines were generated at the Icahn School of Medicine at Mount Sinai. All cell lines were tested for mycoplasma using a mycoplasma detection kit (InvivoGen). NALM6, and REH parental cells were exposed to HD37-dgRTA anti-CD19 immunotoxin (11) at IC_10_ (5 × 10^–13^ M) for 7 days. The dose was escalated as shown in Figure 1A for a total of 30 days of treatment. Once resistant cells were established and in culture, they were treated every 2–3 days with immunotoxin at the IC_75_ dose to maintain resistance.

### Viability assays.

Cells (10,000 cells) were plated in triplicate in 100 μL media (immunotoxin was included in media for resistant cell lines) for 72 hours. CellTiter Blue reagent (Promega) was added to each well as per the manufacturer’s instructions (20 μL reagent/100 μL media). Cells were incubated at 37°C for 1 hour and then read by a FLUOstar Omega plate reader (BMG LABTECH). Statistical analysis of viability assays was performed using GraphPad Prism software as a nonlinear regression inhibitor concentration versus a normalized response on a variable slope. Inhibitory concentration values within a 95% CI were generated as well as the SD.

### scRNA-Seq.

scRNA-Seq was performed at the Icahn School of Medicine at Mount Sinai. NALM6-P and NALM6-R cell lines were sequenced on the 10x Genomics Chromium platform using 3′ v2 chemistry, and raw outputs were generated by running 10x Genomics Cell Ranger software. These outputs were merged, also using the Cell Ranger Software, to allow for joint analysis of the cell lines using 10x Genomics Loupe browser software. A total of 11,217 cells were analyzed. Additional bioinformatics analysis was performed using R software.

### Bulk RNA-Seq.

Total RNA was isolated using the QIAGEN RNeasy kit, and quality was analyzed using the Agilent 2100 Bioanalyzer. Libraries were generated and read by the Illumina HiSEQ 2000 as previously described in Bhattacharyya et al. (20). Sequencing was performed by the Epigenomics Shared Facility at the Albert Einstein College of Medicine Center for Epigenomics.

### ATAC-Seq.

A suspension of 50,000 cells from REH parental and REH resistant cell lines was harvested and resuspended in cold lysis buffer. Lysates were incubated with transposon reaction mixture at 37°C. ATAC-Seq was performed as previously described in Bhattacharyya et al. (20). Sequencing was performed by the Epigenomics Shared Facility at the Albert Einstein College of Medicine Center for Epigenomics.

### Flow cytometry.

Flow cytometry on cell lines was performed using the Flow Cytometry Core at the Albert Einstein College of Medicine. Analysis was performed using the following antibodies: CD19 (MHCD1928, Thermo Fisher Scientific), CD22 (302506, BioLegend), Zombie NIR (423105, BioLegend), and IgG1 (25471480, Thermo Fisher Scientific).

Flow cytometric analysis was uniformly performed on all patient specimens by the NCI’s Flow Cytometry team. Each patient had at least 2 assessment time points available for analysis. NCI specimens were processed within 12 hours of collection and stained with a panel of antibodies as previously described (21). RBC lysis of whole blood lysis was performed using ammonium chloride prior to staining for 30 minutes at room temperature with a panel (Supplemental Table 1) of antibodies (antibody concentration according to the manufacturer’s recommendations). At least 1 million cells were acquired per tube using an 8-color multiparametric approach on a 3-laser FACSCanto II (BD Biosciences) with FACSDiva 6.1.1 software and analyzed by FCS Express software (DeNovo Software). B-ALL cells were identified and distinguished from both mature B cells and precursor B cells/hematogones on the basis of expression patterns of multiple antigens that included, but were not limited to, CD19, CD10, CD20, CD34, CD38, CD45, CD22, and CD24. We used a previously published flow cytometry strategy specifically designed for disease detection in patients who had received CD19-targeted therapy (22). Inherently CD19^–^ cells within the specimen, including T cells and monocytes, were used as an internal negative control (see below).

### Quantification of CD19 and CD22 expression in patient samples.

The antibody bound per cell (ABC) was determined as previously described (21) for anti-CD19 (clone SJ25C1) and anti-CD22 (clone S-HCL-1, BD Biosciences) on leukemic blasts. This was done using saturating concentrations of antibody and the BD Biosciences QuantiBRITE system (QuantiBRITE standard beads and QuantiCALC software) for fluorescence quantitation. The ABC value represents the mean value of the maximum capacity of each cell to bind the antibody. QuantiBRITE PE beads are precalibrated standard beads containing known numbers of PE molecules bound per bead. QuantiBRITE beads were acquired on a FACSCanto II (BD Biosciences) on the same day at the same instrument settings as the individual patient specimens. A standard curve comparing the geometric mean of fluorescence to known PE content of the Quanti-BRITE beads was constructed using QuantiCALC software (BD Biosciences). The regression analysis, slope, intercept, and correlation coefficient were determined. By gating on the basis of immunophenotype using other antibodies in the panel specific for the abnormal lymphoblasts, the data from abnormal B lymphoblasts were isolated, and the geometric mean fluorescence of CD19 and CD22 staining was determined. The ABC values were generated from the measured geometric mean fluorescence of the gated cells using the QuantiBRITE standard curve. T cells served as internal negative controls for both CD19 and CD22 staining. The negative ABC range was used to confirm positivity versus negativity; blasts with CD19 or CD22 staining less than or equal to that of T cells were considered negative for CD19 or CD22 (23).

### Immunoblotting.

Total protein lysates were obtained from 1–2 million cells by lysing the samples (1% NP-40 lysis buffer 20 mmol/L Tris-HCl, pH 7.5; 1 mmol/L EDTA; 150 mmol/L NaCl [1% NP-40], containing phosphatase inhibitor cocktails 2 and 3 and protease inhibitors [MilliporeSigma]) for 30–45 minutes at 4°C. Cells in the treatment conditions were either treated with ibrutinib or trametinib (both from Selleck Chemicals) for 1 hour. An equal amount of protein was prepared by calculating the protein concentration using Bradford reagent (Bio-Rad), and 40 μg protein was resolved on 10%–12% SDS-PAGE gel followed by transfer onto a PVDF membrane (MilliporeSigma). Western blot analysis was performed with the following antibodies: ERK (4695, Cell Signaling Technology), p-ERK (4377, Cell Signaling Technology), BTK (8547S, Cell Signaling Technology), p-BTK (87141S, Cell Signaling Technology), CD19 (90176S, Cell Signaling Technology), and β-actin (sc1615 HRP, Santa-Cruz Biotechnology). Ratio analysis was done using Bio-Rad Image Lab software.

### Mass cytometry (CyTOF).

Antibodies were either purchased preconjugated from Fluidigm or purchased, purified, and conjugated in-house using MaxPar X8 Polymer kits (Fluidigm) according to the manufacturer’s instructions (Supplemental Table 1). Cells were washed with cell-staining buffer (PBS with 0.2% BSA and 0.02% NaN3), labeled with Rh103 intercalator (Fluidigm) as a viability dye, and then stained with cell-surface antibodies for 30 minutes on ice. Then cells were then fixed with 1.6% formaldehyde for 10 minutes and permeabilized with ice-cold methanol and further stained with antibodies against intracellular phosphoprotein targets for 30 minutes on ice. The CyTOF antibodies used are listed in Supplemental Table 1. The samples were then washed and incubated in 0.125 nM iridium intercalator (Fluidigm) diluted in PBS containing 2% formaldehyde for 30 minutes and stored at 4°C until acquisition. Immediately prior to acquisition, samples were washed once with PBS and once with deionized water and were then resuspended at a concentration of 1 million cells per milliliter in deionized water containing a 1:20 dilution of EQ 4 Element Beads (Fluidigm). The samples were acquired on a CyTOF instrument (Fluidigm) equipped with a SuperSampler fluidics system (Victorian Airships) at an event rate of fewer than 500 events per second. After acquisition, the data were normalized using the bead-based normalization algorithm in the CyTOF software (Fluidigm). Barcodes were deconvoluted using the Fluidigm debarcoding software. The data were gated to exclude residual normalization beads, debris, dead cells, and doublets for subsequent clustering and high-dimensional analyses. Normalized and debarcoded data were uploaded to Cytobank (24) for final analysis.

### Statistics.

Statistical analyses were performed using GraphPad Prism 8.4.3 (GraphPad Software) for Mac OS. A 2-tailed Student’s *t* test and the Wilcoxon signed-rank test were used to determine statistical significance. Data represent the mean ± SD. Significance was defined as a *P* value of less than 0.05.

### Study approval.

Patient samples used for this study were obtained with written informed consent under approval of the Albert Einstein College of Medicine IRB (protocol 2005-536) and the National Cancer Institute (NCI), NIH. All patients or guardians gave informed consent for study participation and/or sample collection. Pediatric patient samples were collected as part of the NCI clinical trials NCT01593696 and NCT03448393 (ClinicalTrials.gov).

### Data availability.

Sequences from scRNA-Seq experiments have been deposited in the NCBI’s BioProject database (PRJNA1073528). ATAC-Seq and RNA-Seq sequences have been deposited in the NCBI’s Gene Expression Omnibus database (GEO GSE255305 and GSE255306, respectively). All supporting data for the figures and supplemental data are provided in the Supporting Data Values file.

## Author contributions

S Aminov wrote the manuscript, designed and conducted experiments, performed data analyses, and revised the manuscript. OG, VP, CP, BY, HWW, SS, SGM, VVL, CS, S Aluri, and MS designed and conducted experiments and performed data analyses. DTM and SP designed and conducted single-cell experiments. AS, YW, BA, SKB, MG, IM, AS, WM, and US designed and conducted experiments with primary samples and provided patient samples. KP performed bioinformatics analysis. JDB, NNS, and AV wrote the manuscript, analyzed data, and provided reagents. RAS designed and conducted 7 experiments with primary samples and provided patient samples.

## Supplementary Material

Supplemental data

Unedited blot and gel images

Supporting data values

## Version 1 02/20/2024

In-Press Preview

## Version 2 04/15/2024

Electronic publication
