# Supplementary material for: Immunotherapy-resistant acute lymphoblastic leukemia cells exhibit reduced CD19 and CD22 expression and BTK pathway dependency
Source: J Clin Invest. 2024 Feb 20;134(8):e175199. doi: 10.1172/JCI175199 (PMC11014656; doi:10.1172/JCI175199)
Supplement: Supplemental data [file jci-134-175199-s099.pdf]

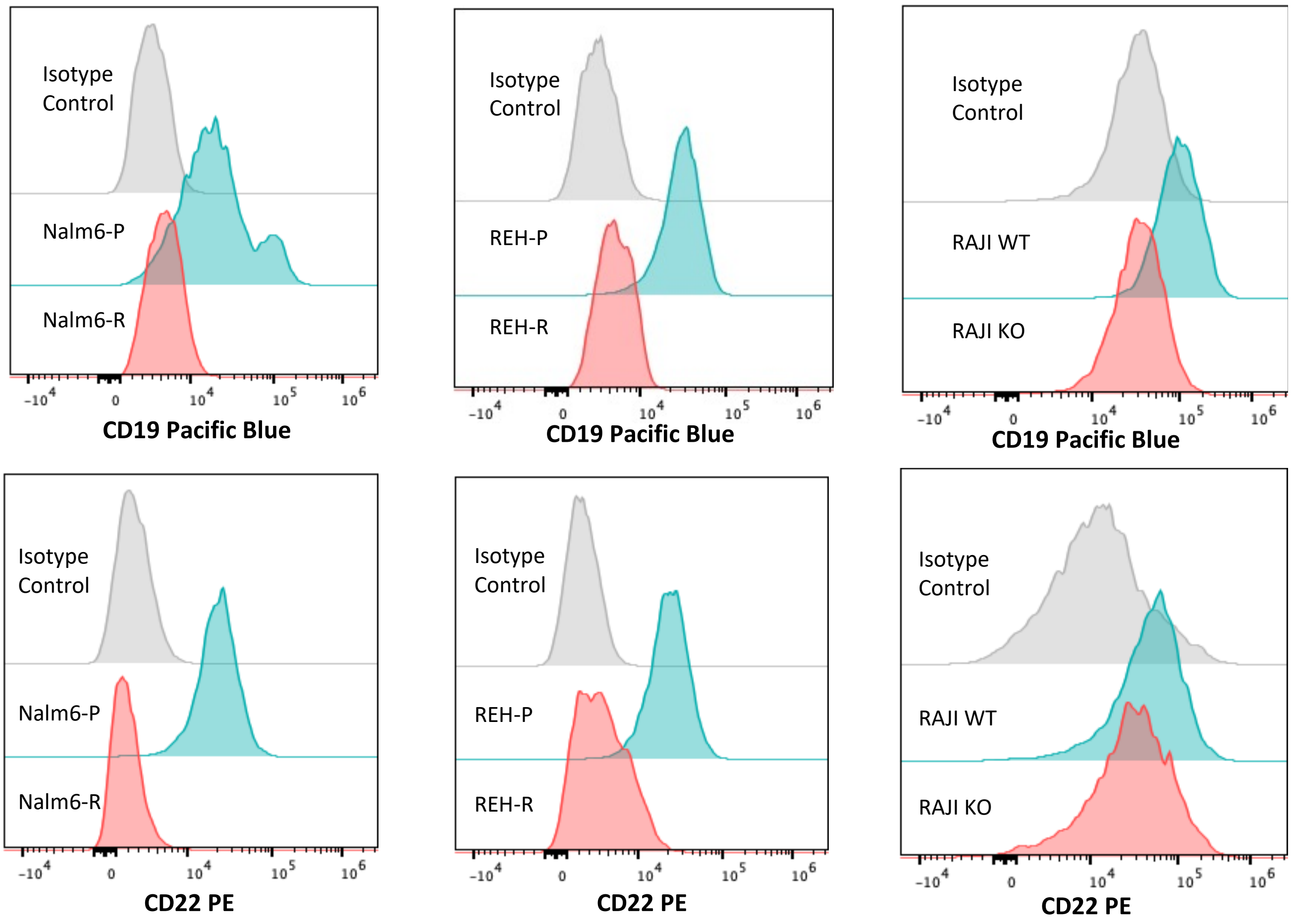


**Supplementary Fig 1:** CD19 and CD22 flow cytometry with isotype controls for Nalm6 and REH parental and resistant cells as well as RAJI WT and CD19 KO cells.

| CyTOF Mass Cytometry Panel | | | |
|---|---|---|---|
| **Target** | Clone | Source | Isotope |
| **CD19** | HIB19 | Biolegend | Nd142 |
| **CD20** | 2H7 | Biolegend | Sm147 |
| **p4E-BP1** | 236B4 | Cell Signaling | Sm149 |
| **pp38** | D3F9 | Fluidigm | Gd156 |
| **CD5** | UCHT2 | Biolegend | Gd158 |
| **pMAPKAP2** | 27B7 | Fluidigm | Tb159 |
| **pPLCg2** | K86–689.37 | Fluidigm | Dy162 |
| **pCREB** | 87G3 | Fluidigm | Ho165 |
| **CD3** | UCHT1 | Biolegend | Er168 |
| **CD22** | HIB22 | Biolegend | Tm169 |
| **pERK1_2** | D13.14.4E | Fluidigm | Yb171 |
| **HLADR** | L243 | Biolegend | Yb174 |
| **CD21** | Bu32 | Biolegend | Yb176 |

**Supplementary Table 1:** Antibodies used for CyTOF Mass Cytometry

| | Molecules in Network | Score | Focus Molecules | Top Diseases and Functions |
|---|---|---|---|---|
| 1 | ARF5,ATXN10,CHD4,CLEC11A,CTBP1,DDRGK1,DHX9,EIF3A,EIF3B,EIF3H,FAM3C,HNRNPA1,HNRNPU,KCTD17,KRT10,LARP7,MDM2,MRN,NAA38,NTHL1,POP4,PPIA,PPIB,PRDX4,PRPF8,Rac,RPL5,RPS11,RPS13,RPS15,RPS4X,SFPQ,SSB,TKT,TPGS1 | 50 | 33 | [Infectious Diseases, Organismal Injury and Abnormalities, RNA Post-Transcriptional Modification] |
| 2 | 47S pre-rRNA:SSU processome,AATF,AFF3,AKAP,AKAP12,AKAP17A,BMS1,C7orf50,CAPNS1,CCDC85B,DDX10,DDX24,DDX54,DRG1,ERP29,GTPBP4,HMG20B,IFRD2,LRRCC1,LYAR,NOP53,NOP56,p85 (pik3r),RPF1,RPL35,RPL8,RPS14,RPS8,RSL1D1,SRP14,SRP72,SSU Processome,TTF1,WDR46,ZNF207 | 45 | 31 | [Cell Cycle, Cell-To-Cell Signaling and Interaction, Connective Tissue Disorders] |
| 3 | AHCYL1,ATP5MJ,CAMK2,CORO1A,cytochrome C,DUT,EEF1B2,EEF1D,FDX1,GCAT,HSD17B10,HSPD1,ITM2B,ITPR,LAMTOR5,MAP1B,Mpt pore,MT-CO1,MTCH2,NOPCHAP1,NUDCD2,OAT,PHGDH,PPIF,RRAGD,SARS1,SLC25A5,SLC25A6,TIMM10,TOMM40,TRABD,UXS1,VARS1,VDAC2,VDAC3 | 45 | 31 | [Connective Tissue Disorders, Developmental Disorder, Neurological Disease] |
| 4 | ATG101,ATP5F1A,ATP5F1D,ATP5PB,ATP5PD,ATP5PF,ATPAF1,BAD,COX6C,COX8A,Creb,CYC1,DDX39A,FADS3,HSP90:ATP:STIP1:HSP70:nascent protein,HSP90:STIP1:HSP70:nascent protein,HSP90AB1,HSPA8:LAMP2a multimers,HSPA9,LSM3,NDUFA1,NDUFA12,NDUFAB1,NDUFB2,NDUFB4,NDUFB6,NTAN1,PRKAA,PSMC5,SDHAF1,SH3BGRL3,SOD1,TCL1A,TRPT1,TUFM | 43 | 30 | [Neurological Disease, Organismal Injury and Abnormalities, Skeletal and Muscular Disorders] |
| 5 | 28S ribosomal subunit,28S ribosomal subunit:MTIF3,39S ribosomal subunit,55S ribosome:mRNA:fMet-tRNA,55S ribosome:mRNA:tRNA:MRRF,CHCHD1,COPZ1,GPATCH4,JAK1/2,LCP1,MRPL12,MRPL13,MRPL14,MRPL21,MRPL23,MRPL27,MRPL3,MRPL32,MRPL36,MRPL37,MRPL41,MRPL42,MRPL54,MRPL55,MRPS12,MRPS15,MRPS18C,MRPS36,OXA1L,PTK2B,SPATS2,SSBP2,TMED2,TMED3,TMED9 | 40 | 29 | [Cell Death and Survival, Cell-mediated Immune Response, Cellular Movement] |
| 6 | ADSL,ANP32E,ATAC B,capped, methylated pre-mRNP:CBC,CIRBP,EDF1,EFTUD2,GMDS,HNRNPDL,HNRNPR,KDELR2,LUC7L3,MAT2A,MLLT11,NOSIP,NTPCR,NXF1,2:NXT1::polyadenylated, capped mRNA:CBC:EJC:TREX:SRSF proteins,PAICS,PCBP2,PHB1,polyadenylated, capped mRNA:CBC:EJC:TREX:SRSF proteins,RAB18,RBM10,RBM3,SLC25A1,SNRPB,SON,spliced mRNA:EJC:CBC complex:Alternative splicing factor,Spliced mRNP,SRSF11,SRSF7,TRA2B,TYMS,ZC3H15,ZMAT2 | 40 | 29 | [Nucleic Acid Metabolism, RNA Post-Transcriptional Modification, Small Molecule Biochemistry] |
| 7 | CLEC2B,DHRS7,EMC10,ERGIC2,FAM216A,GGH,LMAN1,LRPAP1,Mek,MFNG,MIEN1,NPC:NXF1,2:NXT1:EJC:CBC:mRNA,nucleoporin-associated Rev:Importin-beta:B23,NUP210,PXDN,RAB10,RAB11A,RAB9A,RHOBTB3,RHOBTB3:ATP:RHOBTB3 interacting proteins,RTN3,RTN4,SDF2L1,SEC61B,SEC62,SPC25,STARD3NL,STIM2,STK32C,SYVN1,TEX264,TMEM230,Ube2-ubiquitin,UBE2J1,Ubiquitin:E2 conjugating enzymes | 40 | 29 | [Cellular Assembly and Organization, Cellular Function and Maintenance, Tissue Development] |
| 8 | Ap1 gamma,ARPP21,CBX5,Cd1,CD81,CDK1/2,CFLAR:LUBAC:Ub:UBE2D3,DDX18,E2F4,EBNA1BP2,FBL,GBP4,GNL3,HNRNPK,MRPL47,NCL,NPM1,POLR1G,RBCK1,RPL17,RPL22,RPL23A,RPL30,RPL38,RPL4,RPL6,RPL9,RPLP0,RPSA,RRS1,SRSF5,TCR,TH2 Cytokine,YBX1,YBX3 | 40 | 29 | [Cancer, Cell Death and Survival, Organismal Injury and Abnormalities] |
| 9 | ACAA2,ACAT1,ACAT2,acetyl-CoA C-acetyltransferase,APOO,C1QBP,CHCHD10,COX14,Cpla2,CXXC5,ECHS1,growth factor,growth factor receptor,IKK (complex),MCUB,MCUR1,MIX23,MRPL11,MRPS26,MRPS7,MTFMT,MTIF3,NDUFA5,NDUFA9,NDUFS3,NDUFV2,NME4,Pdgfr,PRDX3,PYCR2,SHMT2,Tnf (family),TRMT10C,WIPI1,YME1L1 | 38 | 28 | [Hereditary Disorder, Metabolic Disease, Organismal Injury and Abnormalities] |
| 10 | BMI1,BRCA1,CYFIP1,DDIT4,DNMT1,EZH2,GOPC,H2AX,Histone h2a,HK2,HNRNPC,HSF1:HSP90:HDAC6:VCP:PTGES3,IDH2,KPNB1,LCMT1,LDHA,MACROH2A1,MIR101,NOLC1,PARP,PKM,RIF1,SARAF,SNHG7,SNRPD3,TRIM28,Ub-CDC25A,Ub-misfolded nascent CFTR:ERAD,UBC,UQCRC2,VCP,VCP:Misfolded Proteins:HDAC6:HSP90:HSF1,YY1,ZFAS1,ZNF581 | 38 | 28 | [Cell Cycle, Cell Death and Survival, Organismal Injury and Abnormalities] |
| 11 | ACTB,actin/tubulin-bound CCT/TRiC:ADP,BCL11A,BUD31,C12orf57,CASK,CCDC25,CENPF,CFDP1,CPNE1,CTNNAL1,DPY30,GABP,H2BC8,HSP90:ATP:FKBP4:PTGES3:NR3C1,HSP90:ATP:PTGES3:FKBP52:NR3C1:NR3C1 ligands,INO80 alt,IRF2BP2,KIF5B,MCRS1,MET channel:Tip link:USH1C:USH1G:MYO7A:cytoskeleton (IHC),MET channel:Tip link:USH1C:USH1G:MYO7A:cytoskeleton (OHC),NBEAL1,NR3C1,OAZ1,PCBD1,PRCC,SHKBP1,SMARCE1,SRI,SS18L2,TMIE,UCHL5:INO80,UHRF1,VPS35 | 36 | 27 | [Cell Cycle, Cell Morphology, Cellular Development] |
| 12 | ABHD17A,AIMP2,AK2,ASMTL,Basal body:transition zone proteins,BBS/CCT,CCT/TRiC:ADP:non-native tubulin,CCT/TRiC:ATP:unfolded tubulin,CCT2,CCT5,CCT6A,CRMP2-KLC1-Tubulin,DCTN3,DYNC1H1,DYNLL1,ENDOV,GFOD1,LY86,MAP2K1/2,MAPRE1,Misfolded Proteins:HDAC6:Dynein,NUDC,PCM1,PDCL:Partially folded G-protein beta subunit:CCT/TRiC:ADP,PSMA3,STK24,TEDC2,TRMT6,TUBA1A,TUBA1C,TUBB,TUBB2A,TUBB4B,TUBG1,Tubulin alpha/beta | 34 | 26 | [Cancer, Ophthalmic Disease, Organismal Injury and Abnormalities] |
| 13 | 60S ribosomal subunit,APC (complex),ATF4:CEBPB,CEBPG,DDIT3:TRIB3 gene,BOP1,CCNG1,Eif2,ENY2,FKBP11,Mapk,MAZ,MLX,PDCD10,PFK,PHACTR1,PHACTR3,PP1 protein complex group,pyruvate kinase,RIDA,RPL14,RPL21,RPL22L1,RPL24,RPL26,RPL26L1,RPL27,RPL27A,RPL29,RPL36,RPL36A,RPL37,RPL39L,RPLP2,TRIB3,YAP/TAZ,YAP/TAZ-14-3-3 | 32 | 25 | [Endocrine System Disorders, Metabolic Disease, RNA Post-Transcriptional Modification] |
| 14 | 370 kDa subcomplex,AKT1,ALKBH7,ATP5,COX5B,COX7B,Cytochrome bc1,Cytochrome c oxidase core,Cytochrome c oxidase:CO,cytochrome-c oxidase,GC-GCR dimer,GLRX2,ISCU,Mitochondrial complex 1,Mitochondrial Electron Transport Chain,MT-CO3,MT-CYB,MT-ND1,MT-ND2,MT-ND3,MT-ND4L,MT-ND5,MT-ND6,NADH dehydrogenase,NDUFA8,NDUFB10,NDUFB5,NDUFC2,NDUFS4,NDUFS5,Oxphos,REX1BD,TMEM126B,UQCR11,UQCRH | 30 | 24 | [Cell Signaling, Post-Translational Modification, Protein Synthesis] |
| 15 | AKR1A1,Aldose Reductase,ARL1,ATF7IP2,C1orf21,C4orf3,CCAN:DNA:CASC5,CCAN:DNA:CASC5:Mis18,CD53,CENPM,CENPN,CISD1,CISD2,DCXR,DECR2,Eotaxin,glutathione peroxidase,glutathione transferase,GST (complex),GSTK1,GSTO1,H2AC12,ITGB1,Kinetochore:Mad1:MAD2,MGST3,NME3,PEX5S,L:Cargo protein,PEX5S,L:Cargo:PEX13:PEX14:PEX2:PEX10:PEX12,PLD4,QSOX2,SAR1A,SSR3,SSR4,Translocon:TRAP,TRAPPC2L | 30 | 24 | [Carbohydrate Metabolism, Small Molecule Biochemistry, Vitamin and Mineral Metabolism] |
| 16 | CHCHD2,CLK3,DDX17,DDX21,DDX5,DMAC1,EWSR1,GNL3:EBNA1BP2:DDX21:PES1,GUK1,HDL-cholesterol,HNRNPD,HNRNPM,InlB:MonoUb-K,MET dimer:GRB2-1:CBL:CIN85:endophilin,InlB:MonoUb-K,MET:GRB2-1:CBL:CIN85:endophilin:EPS15:HGS:STAM,INTS11,MDK,Microprocessor,PCOLCE2,PDGFRA,Pmca,PNN,PPIase:nsp1,PPIG,RNA polymerase II (2,7):RPAP2:Integrator:LEC:capped pre-snRNA:Initiation factors:snRNA gene,RNA polymerase II (2,7):RPAP2:Integrator:LEC:CBCAP:capped pre-snRNA:Initiation factors:snRNA gene,RNA polymerase II (2,7):RPAP2:Integrator:LEC:Initiation factors:snRNA gene,SF3B2,Sfk,SH3KBP1,SNRNP70,SRRT,THRAP3,TRA2A,VLDLR,ZCCHC17 | 30 | 24 | [Cancer, Organismal Injury and Abnormalities, RNA Post-Transcriptional Modification] |
| 17 | 18SE pre-40S particle,40S Ribosome-eIF3-mRNA-eIF4A-eIF4B-eIF4E-eIF4G,43S Translation Preinitiation,48s,ACAA1,ACAC,amylase,COX20,ECI1,eIF,EIF1,EIF1AY,EIF3,EIF3D,EIF3K,EIF3M,EIF4F,EIF5,Insulin,MPC2,NCBP2AS2,OXLD1,PABP,PDZD11,PHYH,RENBP,Ribosomal 40s subunit,RPP40,RPS21,RPS25,RPS27L,RPS28,RPS29,RPS4Y1,UQCC2 | 28 | 23 | [Cancer, Gene Expression, Protein Synthesis] |
| 18 | AGAP1,Alpha tubulin,ARL2,ATXN2L,BDH2,CCT/TRiC:ADP,CCT/TRiC:ADP:actin,CCT/TRiC:ADP:Sphingosine kinase 1,CCT/TRiC:ATP:actin,CCT4,CCT7,CLINT1,CUTC,CYSTM1,DAAM1,EID1,GNB5:CCT/TRiC:ADP,HDDC3,MCRIP2,NAV1,PDCL3,PDCL:Partially folded G-protein beta 1-4:CCT/TRiC:ADP,PDCL:Partially folded GNB5:CCT/TRiC:ADP,peroxidase (miscellaneous),PFDN5,PRRC2A,RGS7,RGS9,(RGS6,RGS11):GNB5:CCT/TRiC:ADP,RHOA,SMAP1,SMAP2,TMEM167A,Unfolded G-protein beta subunit:CCT/TRiC:ADP,Unfolded G-protein beta subunit:CCT/TRiC:ATP,VBP1,YTHDF2 | 28 | 23 | [Cellular Movement, Post-Translational Modification, Protein Folding] |
| 19 | 3' end cleaved, ligated exon containing,AIMP1,APRT,AUF1 Complexed with Translation and Heat Shock Proteins,BRF1:mRNA Degradation,EGLN,EIF2AK1,EIF4G1,ELF2,EXOSC10:C1D:MPHOSPH6:SKIV2L2:Exosome,EXOSC4,EXOSC5,EXOSC9,exosome,Exosome core,Exosome:SKI,IARS1,KSRP:mRNA Degradation,LTV1,MTORC1,NOB1,PAIP2,PSMC3,RAB4A,Rab5,RBM8A,RHEB,RPS16,RPS20,RPS27,Spliced mRNA:CBC:EJC:TREX,SUMO2,THOP1,TOP2B,TTP:mRNA Degradation | 26 | 22 | [Infectious Diseases, Organismal Injury and Abnormalities, RNA Post-Transcriptional Modification] |
| 20 | B-WICH:rDNA Promoter:KAT2B:KAT2A:EP300,B-WICH:rDNA Promoter:SL1:RNA Pol I,BAZ1B,Calcineurin A,Ck2,Glycogen synthase,Gsk3,H1-3,H1-5,Histone H1,HMGN2,HMGN3,HP1,ITPA,NT5C,NUCKS1,nucleoside-triphosphate diphosphatase,Nucleosome core,NUDT14,NUDT15,NUDT5,PDAP1,Pdgf (complex),peptidylprolyl isomerase,phosphatase,PPP2R3B,PTP4A1,PTPN6,PTPN7,RPS19BP1,SET,SHP,UTP23,WDR83OS,ZBED1 | 24 | 21 | [DNA Replication, Recombination, and Repair, Nucleic Acid Metabolism, Small Molecule Biochemistry] |
| 21 | Ap2,Ap2 alpha,BCR (IgM class),CCDC167,CD164,CD19,CD22,CD79A,CD79B,CSF,DPM3,FAM177A1,GTPBP6,IgD,Immunoglobulin,LEPROTL1,MFSD12,RRP15,SELENOS,SERP1,SUSD3,Tail-anchored protein:ASNA1:ADP,Tail-anchored protein:ASNA1:ADP:WRB:CAMLG,Tail-anchored protein:ASNA1:ATP,Tail-anchored protein:SGTA dimer,Tail-anchored protein:SGTA:BAG6:GET4:UBL4A:ASNA1:ATP,TGS1,TMEM129:DERL1:VIMP:VCP:Ub:UBE2J2, TMEM129:DERL1:VIMP:VCP:UBE2J2,TMEM147,TMEM14B,Ub:UBE2J2,UBE2J2,US11:HLA:TMEM129:DERL1:VIMP:VCP:Ub:UBE2J2,ZDHHC24 | 23 | 20 | [Cancer, Hematological Disease, Immunological Disease] |
| 22 | AASDHPPT,ANKRD39,ARL6IP6,CKS1B,Cyclin E/A:CDK2:CDKN1A,CDKN1B:CUL1:SKP1:SKP2:CKS1B,Cyclin E/A:CDK2:CDKN1A,CDKN1B:CUL1:SKP1:SKP2:CKS1B:3xubiquitin,cytokine,DDB1/DDB2/CUL4A/RBX1,DDB2,Fcgr3,GG-NER incision complex:Open bubble dsDNA,GG-NER pre-incision complex:Open bubble-dsDNA,GPN1,GPN3,GYG1,H2AZ2,MAC,Open bubble-dsDNA:PAR-UV-DDB:TFIIH Core:XPA:PAR-PARP1, PAR-PARP2:CHD1L,OSTC,PFDN2,PHPT1,PITHD1,PKMYT1,Prefoldin-associated actin/tubulin,RNASEH2C,RPS6KA,SNX3,SPINT2,TMEM183A,Ub:XPC:RAD23:CETN2:Distorted dsDNA:UV-DDB,Ub:XPC:RAD23:CETN2:Distorted dsDNA:UV-DDB:PARP1,PARP2,UV-DDB:COP9 Signalosome,UXT,WDR54,XPC:RAD23:CETN2:Distorted dsDNA:UV-DDB | 23 | 20 | [Cell Cycle, Cell Morphology, Reproductive System Development and Function] |
| 23 | CDC40,CTNNBL1,Deadenylated mRNA:Lsm1-7,GINS2,IFITM2,Interferon alpha,ISGF3 bound to ISRE promotor elements,LSM12,LSM2,LSM2-8,LSM4,LSM5,LSM6,LSM7,LY6E,PQBP1,PRP19-CDC5L,PRPF38A,RBMX2,snRNP,snRNP-IgG Immune complex,SNRNP25,SNRPB2,SNRPC,Spliceosomal B Reactome,Spliceosome B,Spliceosome B activated,Spliceosome B star,Spliceosome C,Spliceosome C star,Spliceosome P,SRRM1,U1 snRNP,WBP4,ZNF511 | 23 | 20 | [Cell-To-Cell Signaling and Interaction, RNA Damage and Repair, RNA Post-Transcriptional Modification] |
| 24 | 26s Proteasome,ADRB,BIRC5,CASP4,CDK6,CEBPB,Dynamin,E2F1,E2F1:TFDP1,TFDP2:RRM2 gene,EZH2 Gene:E2F1/2/3:DP1/2,Ferritin,FTL,GSDMD,HISTONE,JTB,LHFPL6,PEX2:PEX10:PEX12:PEX5L:Ub:UBE2D1,2,3:PEX7:PEX13:PEX14,PEX2:PEX10:PEX12:PEX5S,L:Ub:UBE2D1,2,3:PEX13:PEX14,PSMC1,PSMD4,RIPK1:STUB1:Ub:UBE2D3,RNF181:BCL10:Ub:E2,SMC1A,SMC3,SOX4,Stat3-Stat3,Synaptonemal,TCF4,Ub:UBE2D1,UBE2D2,UBE2D3, UBE2E1,UBE2N,Ub:UBE2D3,UBE2D1,2,3:Ubiquitin,UBE2D3,UBE2I,Ubiquitin,VPS28 | 19 | 18 | [Cellular Development, Cellular Growth and Proliferation, Organismal Survival] |
| 25 | ADP:Calcium Bound Sarcomere Protein,ALDH3A2,AP3D1,ATP:Calcium Bound Sarcomere Protein,Calcium Bound Sarcomere Protein,CLTC,DYRK4,EFHD2,EGF-like ligands:EGFR:CBL:Ub-Y53/55-SPRY1/2,EGF-like ligands:Ub-EGFR:CBL,EGF-like ligands:Ub-EGFR:CBL:GRB2,FGFR1,FSH,GPIIB-IIIA,HAX1,HBEGF,HSPA8,Inactive Sarcomere Protein,Laminin (complex),LAMP1,MAP1LC3B,MYH9,P110,PLC gamma,PTK,RAB13,RAB5C,RAC2,Rsk,SLC3A2,STUB1,SYK/ZAP,TTN,Ub-Activated FGFR1 complex:Ub-FRS2,VAV | 19 | 18 | [Cell Morphology, Cellular Assembly and Organization, Cellular Function and Maintenance] |

**Supplementary Table 2:** Ingenuity pathway analysis on N6 scRNAseq showing networks with significant expression changes in N6 parental vs resistant analysis.

| | Molecules in Network | Score | Focus Molecules | Top Diseases and Functions |
|---|---|---|---|---|
| 1 | AIG1,ASGR2,C11orf96,C4orf45,CCDC125,CPNE7,CYBRD1,EBF3,FA2H,FLYWCH1,FXYD6-FXYD2,KIF6,LRRC17,LRRC3B,MLLT10,NFATC1,OTOS,PARVG,PRDM15,SLC16A13,SLC34A2,SPNS3,SS18,SYNDIG1,THAP4,TMCO5A,TMEM120A,TMEM160,TMEM178A,TMEM60,TSPAN18,TTC32,ZNF219,ZNF423,ZNF521 | 37 | 35 | [Cancer, Endocrine System Disorders, Gastrointestinal Disease] |
| 2 | BMI1,CAND1,CAPZB,CLUH,CNTN4,CTSD,DEF6,DPYSL2,ENOX1,HHLA1,HOMER1,INO80D,KIF23,KPNB1,LAMB4,MATR3,MYBBP1A,NHLRC2,PFKM,PLEC,PRPF40A,RCC2,RRBP1,RUVBL1,SERBP1,SPDL1,STAU1,SYNE3,TUBB4A,UFL1,WWP2,YBX3,ZKSCAN1,ZNF385B,ZNF597 | 37 | 35 | [Cancer, Gastrointestinal Disease, Organismal Injury and Abnormalities] |
| 3 | ADAMTSL3,C17orf50,C6orf15,CARHSP1,CCDC33,CD300LG,CELF5,DMRT3,DOP1B,FAM118A,GLRX3,IGFN1,Integrinα,ITGA9,ITGAM,ITGB1BP1,KHDC1,KRTAP13-1,KRTAP5-7,NBPF10 (includes others),NOTCH2NLA/NOTCH2NLB,OLIG3,OXER1,PCED1B,PCSK5,PROP1,S100PBP,SLC43A2,SMOC1,STAC,STK16,TBX22,TRIM42,VGLL3,ZFY | 34 | 34 | [Cell-To-Cell Signaling and Interaction, Connective Tissue Development and Function, Connective Tissue Disorders] |
| 4 | ABL1,AKAP1,BCAT2,CAST,CPEB3,cytochrome C,DNAH7,GPD2,GTF2IRD1,HK1,KASH5,LRRC49,LRRC66,MEGF11,MST1R,MTX2,NAALADL1,PPFIBP1,RAB11A,RAB5C,RAB7A,RTN4,SCAMP1,SEC23IP,SHANK3,SLC25A51,SLC30A9,SLC9A7,STOM,SYNE1,TMEM43,TXN2,VDAC1,VPS33A,ZCCHC2 | 34 | 34 | [Cancer, Organismal Injury and Abnormalities, Reproductive System Disease] |
| 5 | ADARB2,CABYR,CEP112,ERCC6L2,FAM166B,FAM168B,FHL5,FSTL4,GIGYF2,GPATCH2L,Inpp5,INPP5D,INPP5E,LETM2,MAP7D2,MELK,MISP,MORN3,MYOZ3,NEAT1,NEK6,Opsin,PLEKHA5,PNMA2,PPM1H,ROPN1,RTP5,SALL3,SH3PXD2A,STARD13,TEX19,ZCCHC7,ZNF438,ZNF648,ZNF766 | 32 | 33 | [Carbohydrate Metabolism, Lipid Metabolism, Small Molecule Biochemistry] |
| 6 | AMBP,ARRDC3,BRINP2,CMTR1,CSMD1,CUEDC1,DLGAP4,GZMK,Iti,ITIH2,ITIH5,KCNK9,KIF16B,KIF3C,LCN,LCN8,LDLRAD4,MON2,MPP7,NCAPG2,NPLOC4,OBP2B,ORM2,PRSS8,RFFL,SEZ6L,SPIN1,SPINT2,SV2C,SZRD1,TM4SF4,TMEM168,TNFSF13B,TRIM66,TRMT6 | 32 | 33 | [Carbohydrate Metabolism, Developmental Disorder, Small Molecule Biochemistry] |
| 7 | ANKRD11,CERS2,KRAB-ZNF / KAP,ROCK1,SETX,TRIM54,TSG101,VAV,ZFHX3,ZFP69,ZNF100,ZNF155,ZNF175,ZNF230,ZNF287,ZNF33B,ZNF41,ZNF433,ZNF443,ZNF485,ZNF492/ZNF98,ZNF554,ZNF557,ZNF596,ZNF599,ZNF615,ZNF671,ZNF709,ZNF713,ZNF716,ZNF717,ZNF718,ZNF750,ZNF791,ZNF860 | 32 | 33 | [Cancer, Organismal Injury and Abnormalities, Respiratory Disease] |
| 8 | ANXA2,AP3D1,BICD1,CASP1,CCSAP,CDK6,DIPK1C,DYNC1LI1,FGFR4,GFI1,GSDMC,HORMAD2-AS1,Hsp90 (family),HSPA9,INTS1,IRAK1,KIT,MAP4K3,MOXD1,NEDD9,NTRK1,PAK2,RANBP9,SH3KBP1,SYNPO2,TANK,TAX1BP1,TNFRSF8,TNIP2,TNK2,TRAF3IP2,TRAF4,TRAF5,Ubiquitin,ZNF823 | 32 | 33 | [Cancer, Organismal Injury and Abnormalities, Respiratory Disease] |
| 9 | BAIAP2,BAIAP2L1,C6orf132,CAMLG,CEMIP2,EVL,FAM91A1,FHIP2A,FLNC,GAPVD1,HLCS,IRF4,ISGF3 bound to ISRE promotor elements,KIAA1522,LHFPL4,LNPK,NUDT16,PRDM10,PTP4A1,PTP4A2,RAB35,RAP1GDS1,RASL11B,SEC63,SLC25A46,SNX24,SNX29,STBD1,TACC1,TACC2,Tail-anchored protein:ASNA1:ADP:WRB:CAMLG,TEX2,TMSB4,TNFAIP8,TSSK1B | 30 | 32 | [Cancer, Cell Cycle, Cell-To-Cell Signaling and Interaction] |
| 10 | ANKFY1,AP3M1,CAPN13,CCDC88A,CEP19,CTNNA1,DCAF8L2,DLG5,DNAAF10,JMY,KIF11,KPNA4,KSR2,MARK2,MTCL1,NAA11,NS1 homodimer:Importin,PARD3,PDRG1,PHLPP1,PLD1,PPHLN1,PPP6R3,RASSF10,RBM26,REV1,SFSWAP,TJP2,TNKS1BP1,TP53BP2,VE-cadherin-Catenin,YAP/TAZ,ZC3H15,ZC3H4,ZYG11B | 30 | 32 | [Cell Death and Survival, Infectious Diseases, Organismal Injury and Abnormalities] |
| 11 | BGLAP,BMAL1,CNBP,DDX27,DIO2,FCHO1,GPAM,Ifn gamma,IGHV1OR15-1,KLF15,KLF8,LARP1,LARP1B,LIN28A,MAP7,MUC12,NKRF,PPARGC1B,PRDM2,PURPL,RABGAP1L,RIN3,RPL21,RPL36,RPL37A,RPL6,RPLP1,RUNX2:MAF:BGLAP gene,SNORD61,TRIM31,TUT7,VLDL-cholesterol,ZBTB24,ZNF16,ZNF44 | 30 | 32 | [Embryonic Development, Organ Morphology, Organismal Development] |
| 12 | ADAMTSL2,Alpha Actinin,ARHGAP24,B3GNT2,CAPSL,carboxylic ester hydrolase,CES2,DCAF10,EML4,H2BC11,IARS1,KIF2A,LMNB2,LOXL2,LOXL4,NACA,NDST1,NEK9,PAM,PATJ,PKN2,Ras homolog,SCYL1,STOX2,TAGLN2,TECPR1,TKT,TUBB2A,TUBB8,WBP2NL,WDR1,WFIKKN1,ZNF813,ZNF836,ZNF93 | 30 | 32 | [Cellular Movement, Developmental Disorder, Neurological Disease] |
| 13 | ABCB5,ACOX3,ARHGEF10L,B3GNT7,BRF2,BZW2,C3orf18,CAMTA1,COPG1,DYM,EIF2B3,GSDME,MIR124,MLYCD,MOGAT1,MTSS2,NMNAT3,NUDT19,PCGEM1,PEX14,PEX3:PEX19:class I PMP,PEX5S,L:Cargo protein,PEX5S,L:Cargo:PEX13:PEX14:PEX2:PEX10:PEX12,PPP1R15B,PTPRC,PXMP4,SKAP2,SLC27A2,SLC39A11,SNTG1,SRGAP1,SSR3,TMEM38B,TPST2,XPO6 | 28 | 31 | [Lipid Metabolism, Nucleic Acid Metabolism, Small Molecule Biochemistry] |
| 14 | alpha-adrenergic receptor,ARHGAP25,C5orf24,CCT8L2,CELF1,CELF2,CFAP20,CIZ1,CKMT2,DCLK1,FAM110B,FOXN4,GATB,HERPUD1,hnRNP H,MAP2K1/2,MAT2B,MN1,MOB2,NOVA2,PKNOX1,PTBP2,RBM46,RBPMS2,RFX2,RFX3,SFT2D2,SH3BP4,SLC8A1,THUMPD2,THUMPD3,TSPYL5,UCKL1,USP30:MOM proteins,ZFAND2B | 28 | 31 | [Developmental Disorder, Hereditary Disorder, Neurological Disease] |
| 15 | AGK,ANO10,C1orf21,CACHD1,Cathepsin,CTSO,DENND2B,Dgk,DGKD,DGKK,DHRS2,DIP2B,EMP2,GFOD1,KIAA0040,KRAS,Lysosomal Protease,MEAK7,mir-3180,mir-340,MPZL1,MTRR,NAB1,ONECUT2,PLEK2,PRICKLE4,SBF1,SHISA2,SPG7,STON1-GTF2A1L,TMEM161A,USP17L,USP17L22 (includes others),VSTM5,ZNG1B | 28 | 31 | [Cell Death and Survival, Embryonic Development, Organismal Injury and Abnormalities] |
| 16 | apyrase,ATXN8OS,CCDC198,DHRS9,ELFN1,ELFN2,EMB,ERC1,ERC2,FAM9B,FCHSD2,GOLGA1,HSD17B,HSD17B1,KIAA0513,LENG1,LNX2,LZTS1,MICAL3,mir-204,mir-217,NADH2 or NADPH2 1 atom incorporation:oxygen oxidoreductase,NBAS,NCOA7,Nfat (family),PDZRN4,PHYKPL,PRKAR1B,RSRC1,TANC1,TBC1D1,TBC1D5,TEX28,TFDP3,VPS26C | 28 | 31 | [Cellular Development, Hereditary Disorder, Neurological Disease] |
| 17 | 28S ribosomal subunit,28S ribosomal subunit:MTIF3,55S ribosome:mRNA:fMet-tRNA,55S ribosome:mRNA:tRNA:MRRF,AFG3L2,AK4,AURKAIP1,CCSER1,CLPX,COQ8A,ERAL1,FBXL17,GLI4,LARS2,MCAT,MRM1,MRPL33,MRPL46,MRPS27,MRPS5,MTFMT,MTHFD1L,MTIF3,NME4,PCCB,PDCD1,RTN4IP1,SLC4A11,SMIM20,TIMM21,TMEM223,TRAP1,TRMT5,ZKSCAN2,ZNF574 | 28 | 31 | [Hereditary Disorder, Metabolic Disease, Organismal Injury and Abnormalities] |
| 18 | AMPD2,ARID1B,BCHE,BCL7A,BCORL1,C2orf27A/CDRT15P3,CA5A,CHD7,CHD8,CWH43,DIAPH2,ERG,ETV4,EVI5,FAM174B,GKN2,H2BC3,IL7R,KLHL4,LHX2,Osteocalcin,PCAT5,PHACTR4,RAI1,REP15,RESF1,RUNX1:CBFB:SWI/SNF,SH3TC1,SLC45A3,SMARCA2,SMARCB1,SWI/SNF chromatin remodelling,SWI/SNF chromatin remodelling complex:PRMT5:pT5-WDR77,TFCP2L1,VAX2 | 28 | 31 | [Cancer, Gastrointestinal Disease, Organismal Injury and Abnormalities] |
| 19 | ACTG2,ADP:Calcium Bound Myosin Actin,ANXA11,ANXA6,ATP:Calcium Bound Myosin Actin,BRPF3,Calcium Bound Myosin Actin,CALD1,COPS7A,CST5,DCAF12,DCP2,FGGY,GPC1,GPC6,IDH3B,Inactive Myosin Actin Contractile,LGALS9,NHERF2,NSFL1C,OAT,PALS2,PDCL3,PIEZO1,PNPLA1,RORC,SLC1A3,SLC25A10,SLC25A24,SLC7A2,SORBS1,SRP9,TARS1,TOMM40,ZNF491 | 28 | 31 | [Amino Acid Metabolism, Molecular Transport, Small Molecule Biochemistry] |
| 20 | CCL22,CD79A,CD79B,CHST15,CSF1R,EBF1,EBF1:LDB1:LHX2:Intergenic olfactory enhancer:Olfactory Receptor gene,HCK,IgD,IL17R,INPPL1,MKK3/6,OR1D5,OR1E1,OR1E2,OR1J2,OR1M1,OR2F1,OR4C46,OR4C5,OR4E1,OR4F17 (includes others),OR4F21 (includes others),OR5A2,OR5B21,OR6B3,OR6C4,OR6J1,OR6S1,OR6X1,OR8B8,PAX5,PHKG1,SH2B2,VPREB1 | 28 | 31 | [Cell-To-Cell Signaling and Interaction, Cellular Development, Nervous System Development and Function] |
| 21 | 1-acylglycerol-3-phosphate O-acyltransferase,ABHD17C,AGPAT3,amylase,AMZ1,CACNA1E,CAMKV,DCLK2,FIBCD1,GOLT1A,GPAT2,GPM6A,High voltage-activated calcium channel,JCAD,LAMP5,LCLAT1,LMBR1,LPCAT3,MAPT,MBOAT2,MEI1,Microtubule Associated,NHSL1,PHYH,RNF24,SLC22A16,STOX1,SYN2,Synapsin,TMEM104,TRIM3,TSPAN3,TSPAN5,VKORC1L1,ZFYVE28 | 26 | 30 | [Cancer, Connective Tissue Disorders, Developmental Disorder] |
| 22 | ABTB2,ANKLE2,ARAP3,BNIP3,Collagen type VI,EHBP1,ERF,ESRP2,ETV3,EVC2,FNDC3A,IFT B,IFT B*,IFT57,IFT81,KIAA1671,KLHL26,NPAS1,PDXDC1,PLEKHG7,PRELID2,PTRH2,RIN1,SLC18A1,SLC18A2,SLC45A4,SMTN,SPNS2,TCF23,Tgf beta,Timp,TNS4,TRAF3IP1,TTC30B,UBXN6 | 26 | 30 | [Cell-To-Cell Signaling and Interaction, Drug Metabolism, Small Molecule Biochemistry] |
| 23 | AGTRAP,ALS2,ATP10B,CADPS,CYB5A,CYB5B,FAM117B,GALNT9,HMOX1,HPCAL1,ICAM5,IQSEC1,Kcnj,KCNJ1,KCNJ15,KCNJ18,KCNJ2,KCNJ9,MIEF2,NAPA,OGDHL,PHACTR3,PRB1/PRB2,RETREG1,SEC22B,SEC61B,SLC35A3,SPIN4,STX1A,Tail-anchored protein:ASNA1:ADP,Tail-anchored protein:ASNA1:ATP,Tail-anchored protein:SGTA dimer,Tail-anchored protein:SGTA:BAG6:GET4:UBL4A:ASNA1:ATP,TPK1,UGT8 | 26 | 30 | [Cell-To-Cell Signaling and Interaction, Cellular Assembly and Organization, Nervous System Development and Function] |
| 24 | 2x RET:GDNF:GFRA complexes with, without SHC1:GRB2-1:GAB1,GAB2:PTPN11,47S pre-rRNA:SSU processome,ARHGEF4,BBX,CENPU,CHIC1,CLIP2,ETV6,FARP1,FGFBP1,FOXB1,GAB2,IL15:IL15RA:IL2RB:JAK1:IL2RG:JAK3:SHC1:GRB2:GAB2,KNOP1,KRR1,NAT10,NEPRO,NIFK,NOL10,NOP53,NSRP1,PARN,PIP4K2A,PPIL4,RBM28,Rnr,RPL26L1,RRP8,SNORD3A,SSU Processome,SUGP2,URB2,UTP14A,UTP25,WDR36 | 26 | 30 | [Cellular Growth and Proliferation, Connective Tissue Disorders, Embryonic Development] |
| 25 | ACSL3,AHNAK,ALPP,ATP2A1,ATXN10,BRF1,CCDC47,CCDC8,CD3 group,CDK4/6,CENPV,CYB5R1,DERL1,EMC2,ENPP7,EPHX1,EPHX3,epoxide hydrolase,ERGIC1,LIG3,LTA4H,MAC,MARCHF5,NIBAN2,NIPA1,NOMO1 (includes others),PANX1,PLC gamma,POR,PYGB,RHBDD1,SDS,SLC25A26,STT3B,UBIAD1 | 26 | 30 | [Cell Death and Survival, Renal and Urological System Development and Function, Renal Necrosis/Cell Death] |

**Supplementary Table 3:** Ingenuity pathway analysis on REH ATACseq showing networks with significant expression changes in REH parental vs resistant analysis.

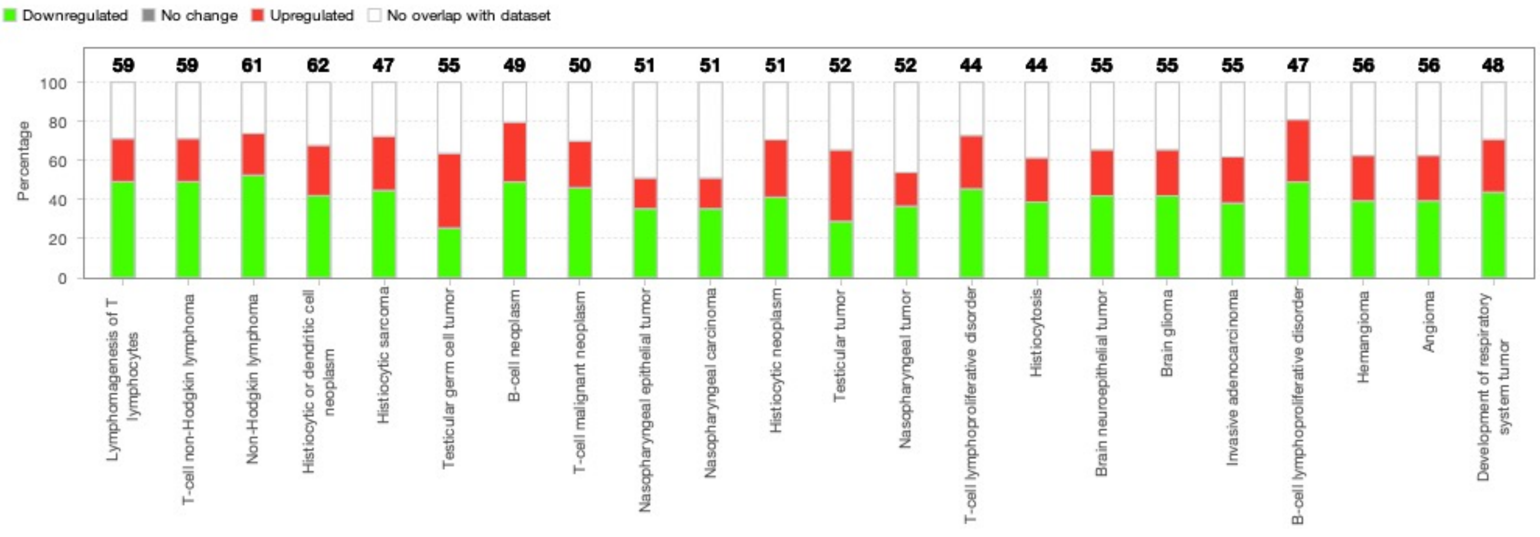


**Supplementary Fig 2:** Ingenuity pathway analysis on N6 scRNAseq showing up and down regulation of genes in disease pathways.

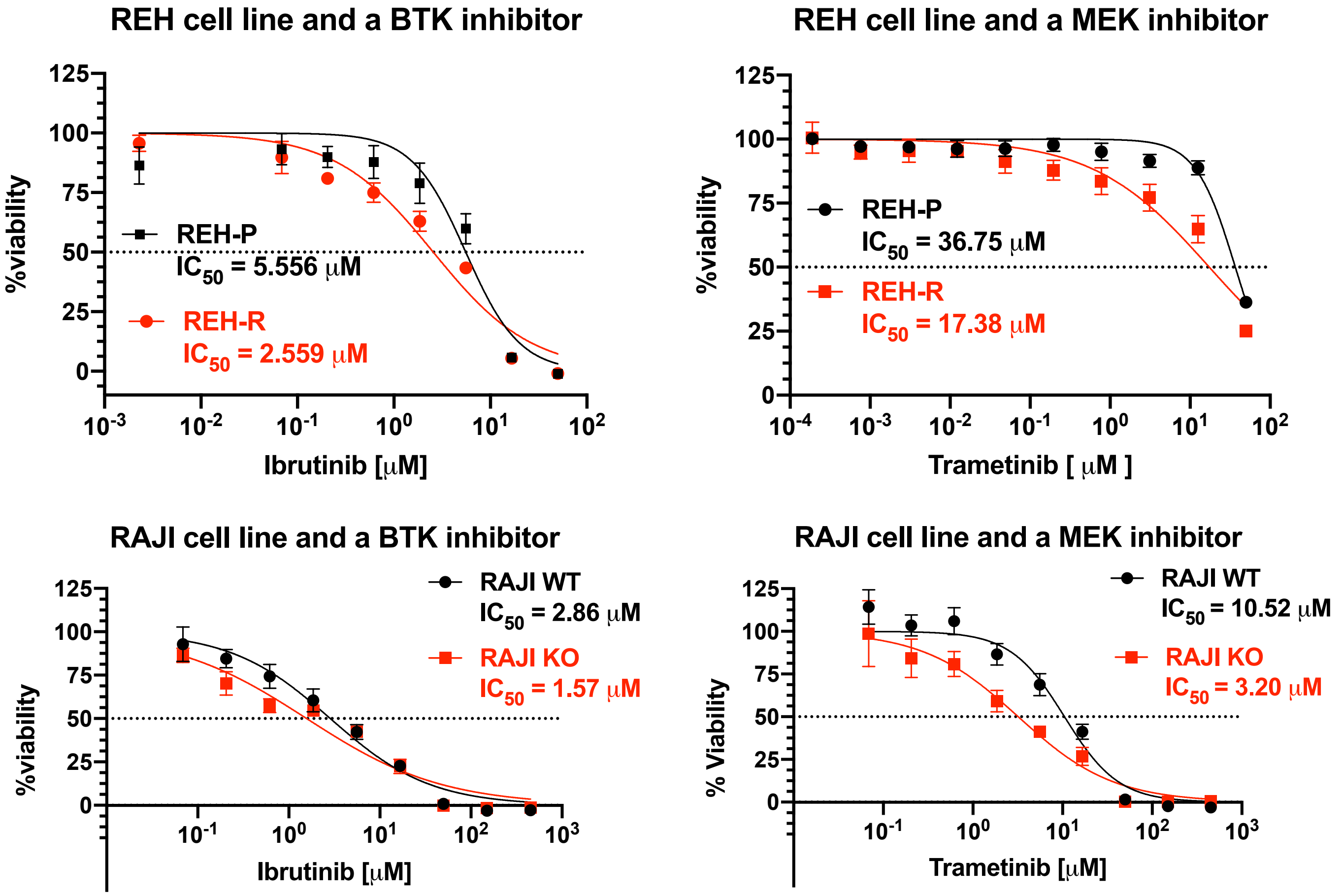


**Supplementary Fig 3:** Resistant REH cells (n=4) and RAJI CD19 KO cells (n=5) were more sensitive to BTK and MEK inhibition than parental and WT cells, respectively.

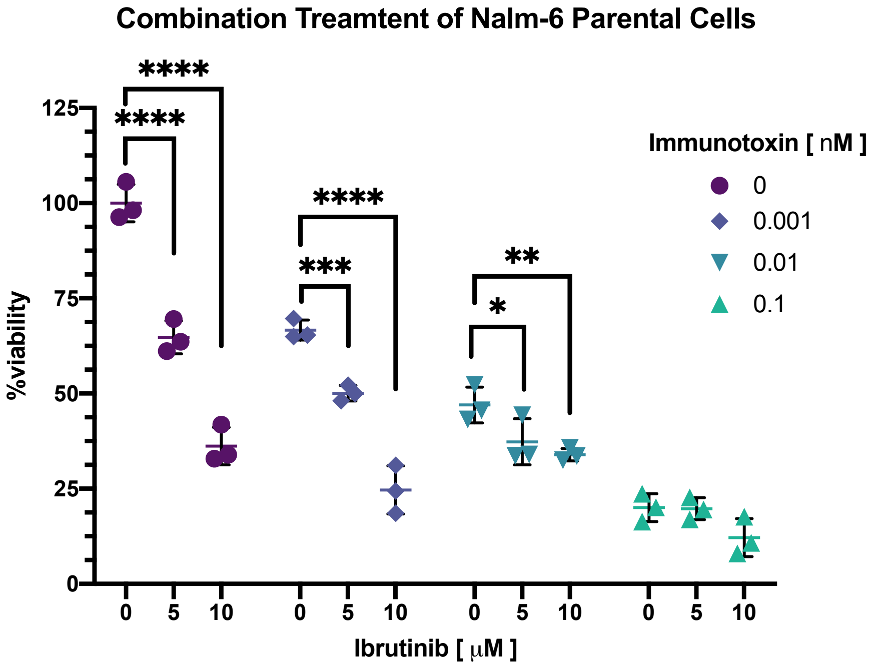


**Supplementary Fig 4:** Parental Nalm-6 cells treated with anti-CD19 immunotoxin and BTK inhibitor, ibrutinib, alone in combination show significant loss of proliferation in combination treated cells (n=3). Significance was determined using 2-way Anova.
