# Supplementary material for: Immunotherapy-resistant acute lymphoblastic leukemia cells exhibit reduced CD19 and CD22 expression and BTK pathway dependency
Source: J Clin Invest. 2024 Feb 20;134(8):e175199. doi: 10.1172/JCI175199 (PMC11014656; doi:10.1172/JCI175199)
Supplement: Unedited blot and gel images [file jci-134-175199-s100.pptx]

## Slide 1
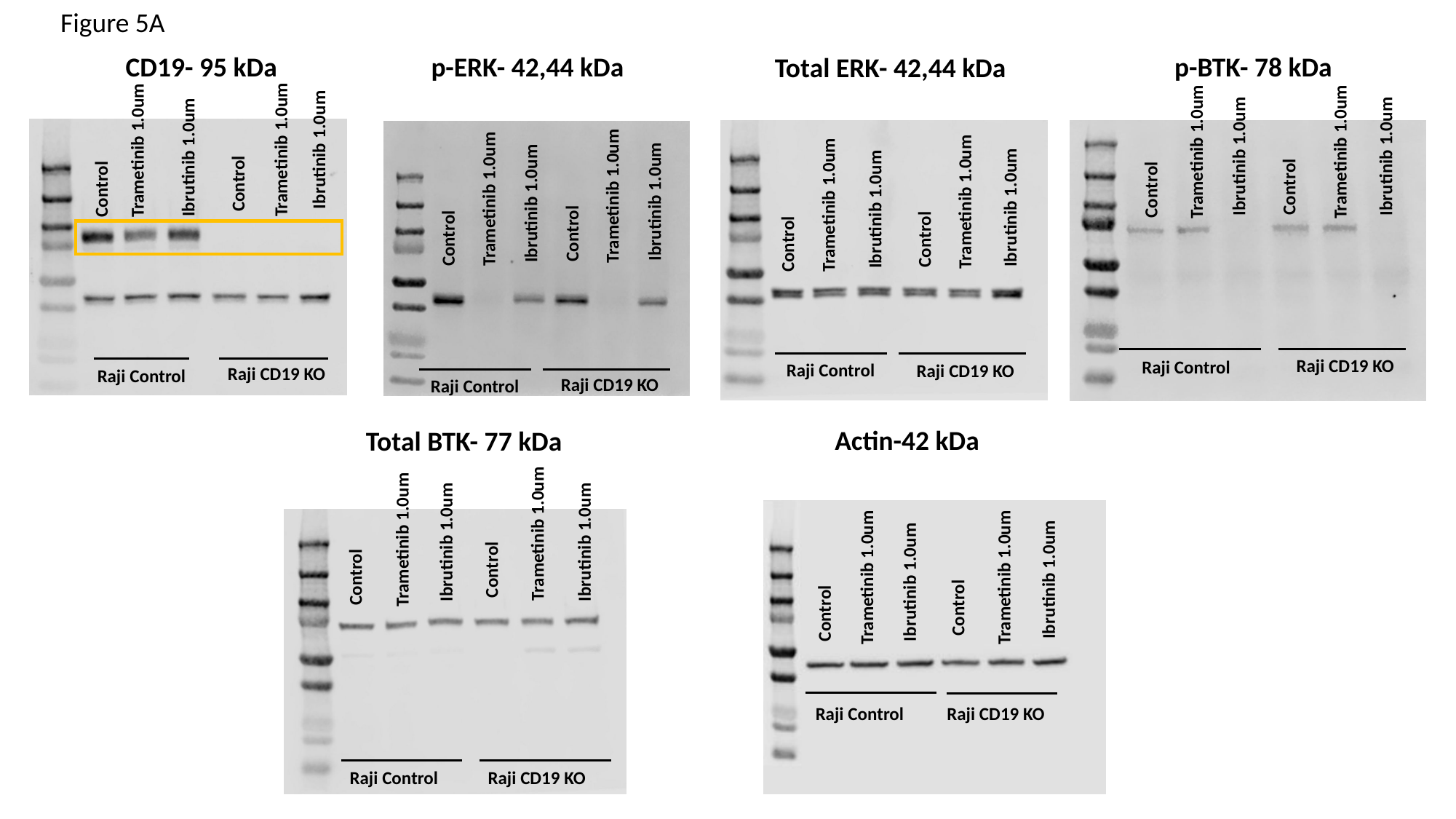

Figure 5A
p-BTK- 78 kDa
CD19- 95 kDa
p-ERK- 42,44 kDa
Total ERK- 42,44 kDa
 Trametinib 1.0um
 Trametinib 1.0um
 Ibrutinib 1.0um
 Ibrutinib 1.0um
Control
Control
Raji CD19 KO
Raji Control
 Ibrutinib 1.0um
 Trametinib 1.0um
 Trametinib 1.0um
 Ibrutinib 1.0um
Control
Control
Raji CD19 KO
Raji Control
 Trametinib 1.0um
 Trametinib 1.0um
 Ibrutinib 1.0um
 Ibrutinib 1.0um
Control
Control
Raji CD19 KO
Raji Control
 Trametinib 1.0um
 Trametinib 1.0um
 Ibrutinib 1.0um
 Ibrutinib 1.0um
Control
Control
Raji Control
Raji CD19 KO
Actin-42 kDa
Total BTK- 77 kDa
 Trametinib 1.0um
 Ibrutinib 1.0um
 Trametinib 1.0um
 Ibrutinib 1.0um
Control
Control
Raji Control
Raji CD19 KO
 Trametinib 1.0um
 Ibrutinib 1.0um
 Trametinib 1.0um
 Ibrutinib 1.0um
Control
Control
Raji Control
Raji CD19 KO

## Slide 2
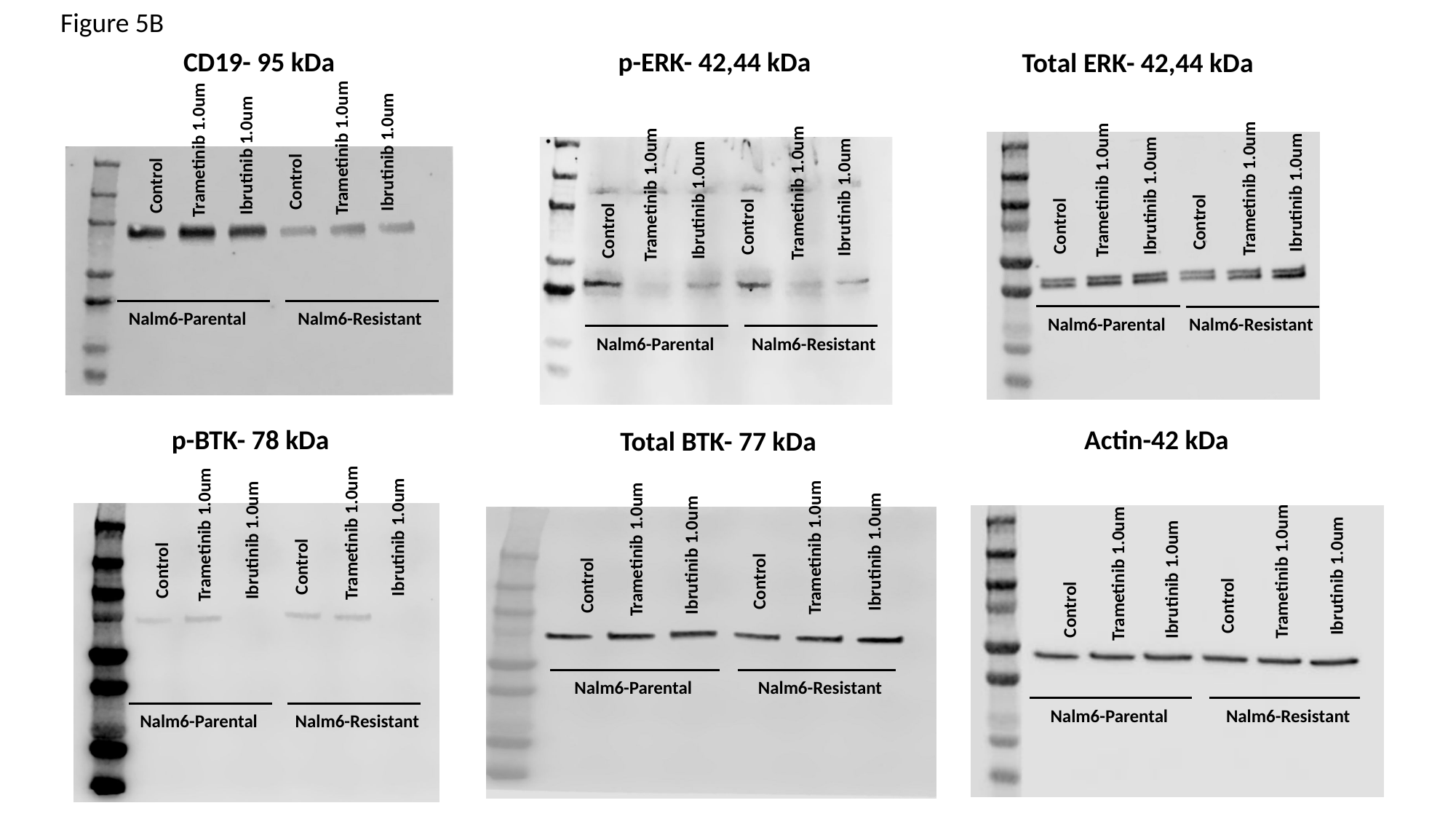

Figure 5B
p-ERK- 42,44 kDa
CD19- 95 kDa
Total ERK- 42,44 kDa
 Trametinib 1.0um
 Ibrutinib 1.0um
 Trametinib 1.0um
 Ibrutinib 1.0um
Control
Control
Nalm6-Parental
Nalm6-Resistant
 Trametinib 1.0um
 Ibrutinib 1.0um
 Trametinib 1.0um
 Ibrutinib 1.0um
Control
Control
Nalm6-Parental
Nalm6-Resistant
 Trametinib 1.0um
 Ibrutinib 1.0um
 Trametinib 1.0um
 Ibrutinib 1.0um
Control
Control
Nalm6-Parental
Nalm6-Resistant
p-BTK- 78 kDa
Actin-42 kDa
Total BTK- 77 kDa
 Trametinib 1.0um
 Ibrutinib 1.0um
 Trametinib 1.0um
 Ibrutinib 1.0um
Control
Control
Nalm6-Parental
Nalm6-Resistant
 Trametinib 1.0um
 Ibrutinib 1.0um
 Trametinib 1.0um
 Ibrutinib 1.0um
Control
Control
Nalm6-Parental
Nalm6-Resistant
 Trametinib 1.0um
 Ibrutinib 1.0um
 Trametinib 1.0um
 Ibrutinib 1.0um
Control
Control
Nalm6-Parental
Nalm6-Resistant
